# Erinacine C Activates Transcription from a Consensus ETS DNA Binding Site in Astrocytic Cells in Addition to NGF Induction

**DOI:** 10.3390/biom10101440

**Published:** 2020-10-14

**Authors:** Monique Rascher, Kathrin Wittstein, Barbara Winter, Zeljka Rupcic, Alexandra Wolf-Asseburg, Marc Stadler, Reinhard W. Köster

**Affiliations:** 1 Division of Cellular and Molecular Biology, Zoological Institute, Technische Universität Braunschweig, Spielmannstraße 7, 38106 Braunschweig, Germany; m.rascher@tu-braunschweig.de (M.R.); b.winter@tu-braunschweig.de (B.W.); alex.wolf@tu-braunschweig.de (A.W.-A.); 2 Department Microbial Drugs, Helmholtz Centre for Infection Research GmbH and Institute of Microbiology, Technische Universität Braunschweig, Inhoffenstraße 7, 38124 Braunschweig, Germany; Kathrin.Wittstein@helmholtz-hzi.de (K.W.); Zeljka.Rupcic@helmholtz-hzi.de (Z.R.); 3 German Centre for Infection Research (DZIF), Technische Universität Braunschweig, Partner Site Hannover-Braunschweig, 38124 Braunschweig, Germany; 4 Institute of Microbiology, Technische Universität Braunschweig, Spielmannstraße 7, 38106 Braunschweig, Germany

**Keywords:** *Hericium*, cyathane diterpenoid, erinacine, nerve growth factor, brain derived neurotrophic factor, neurotrophin, ETS signaling

## Abstract

Medicinal mushrooms of the genus *Hericium* are known to produce secondary metabolites with homeostatic properties for the central nervous system. We and others have recently demonstrated that among these metabolites cyathane diterpenoids and in particular erinacine C possess potent neurotrophin inducing properties in astrocytic cells. Yet, the signaling events downstream of erinacine C induced neurotrophin acitivity in neural-like adrenal phaeochromocytoma cells (PC12) cells have remained elusive. Similar, signaling events activated by erinacine C in astrocytic cells are unknown. Using a combination of genetic and pharmacological inhibitors we show that erinacine C induced neurotrophic activity mediates PC12 cell differentiation via the TrkA receptor and likely its associated PLCγ-, PI3K-, and MAPK/ERK pathways. Furthermore, a small library of transcriptional activation reporters revealed that erinacine C induces transcriptional activation mediated by DNA consensus binding sites of selected conserved transcription factor families. Among these, transcription is activated from an ETS consensus in a concentration dependent manner. Interestingly, induced ETS-consensus transcription occurs in parallel and independent of neurotrophin induction. This finding helps to explain the many pleiotropic functions of cyathane diterpenoids. Moreover, our studies provide genetic access to cyathane diterpenoid functions in astrocytic cells and help to mechanistically understand the action of cyathanes in glial cells.

## 1. Introduction

Medicinal mushrooms have been studied for neurotrophin inducing metabolites for about 25 years, and especially the genus *Hericium* has raised particular attention [1,2,3]. Extracts from both, basidomes and mycelial cultures of *Hericium erinaceus* are known to induce neurotrophin expression. Furthermore, we and others have recently revealed that isolated cyathane diterpenoids are able to mediate this neurotrophin-inducing activity of *Hericium* extracts [4,5,6,7,8]. Induction of neurotrophin expression by these cyathane diterpenoids does not occur in neuronal cells directly but in astrocytic cells. Among these compounds, erinacine C appeared particularly interesting as this substance was able to induce both Nerve Growth Factor β (NGF) and Brain-Derived Neurotrophic Factor (BDNF) expression in the astrocytoma cell line 1321N1. Yet, the cascades downstream of neurotrophin induction by erinacine C that are likely mediated via the high-affinity Tropomyosin receptor kinase A (TrkA) in PC12 cells have not been identified so far.

Despite inducing neurotrophin expression, it has also been reported that erinacine C exerts anti-neuroinflammatory activity by altering transcription processes in microglia [8]. In addition, erinacines and other compounds from *Hericium erinaceus* have been shown to act as antidepressants [9], to counteract neurodegeneration [10,11], and to promote neurogenesis [12,13]. These many activities of erinacine C suggest that this compound affects transcriptional processes of prominent signal transduction pathways in astrocytic cells beyond neurotrophin induction.

Importantly, the influence of erinacine C in altering expression levels and profiles in astroglial cells directly have not been investigated so far. Clearly a first step towards understanding the mechanisms of action for the activity of erinacine C is needed. This could be guided by analyzing several signaling pathways and their DNA-binding transcription factors including those that are known to act upstream of *ngf* expression [14,15]. We have therefore set out to elucidate erinacine C mediated signal transduction events by analyzing altered transcriptional processes in erinacine C treated astroglial cells. These studies are aimed at providing genetically encoded tools responsive to erinacine C activity and to broaden the understanding of erinacine C induced expression changes in astrocytic cells.

## 2. Materials and Methods

### 2.1. Plasmid Construction

All expression vectors were generated with the help of the pCS2+-vector backbone in which transgene expression is driven by a CMV promoter [16].

pCS-hETS-TA2, -TA4, -KRAB: to amplify the DNA binding domain of human ETS1, RT-PCR was performed with cDNA isolated from 1321N1 cells and the following primers: 5′-TATATCGATCCACCATGGGATCAAGATCGCCAAAAAAGAAGAGAAAGGTGATGGACTATGTGCGGGACCGTGCTGACCTCAATAAGG-3′ and 5′-ATAGTCGACCCCAGCAGGCTCTGCAGGTCACACACAAAGCGGTAC-3′. The obtained cDNA was cloned as *Cla*I/*Sal*I-fragment into pCS-KalTA2, pCS-KalTA4, and pCS-KalKRAB to replace the Gal4 DNA binding domain [17,18,19].

pCS-dnTrkA-EGFP: the N-terminus of rat *trkA* (AA1-477) containing the extracellular and transmembrane domain was amplified from cDNA of PC12 cells and the following primers: 5′-TATGAATTCCACCATGCTGCGAGGCCAGCGGCACGGGCAGCTGGGTTGGCATC-3′ and 5′-ATAGTCGACCCACCCAGTGTCATGAAGTGTAGGGACATGGCCAGCCCATCCTCTG-3′. The introduced *Sal*I-site was used for in frame cloning of a mCitrine cDNA containing an ER exit signal (FCYENEV) at its C-terminus using the following primers: 5′-TATGTCGACAGGCGGAGGCGGAAGCATGGTGAGCAAGGGCGAGGAGCTGTTCACCG-3′ and 5′-ATATCTAGATCTACACTTCGTTCTCGTAGCAGAACTTGTACAGCTCGTCCATGCCGAGAGTGATC-3′.

pCS-FynmCitrine: the myristinylation signal of Fyn kinase (MGCVQCKDKEATKLTST) was added to the N-terminus of mCitrine by PCR.

The Renilla luciferase expression construct used for cotransfections with Firefly luciferase constructs to correct for different transfection efficiencies was generated from pRL-SV40 (Promgea, Inc., Madison, WI, USA). The simian virus 40 (SV40) enhancer/promoter (*Hind*III/*Bgl*II-blunt) was replaced by a SV40 enhancer/tk promoter (*Hind*III/*Xba*I-blunt) fragment.

Luciferase reporter constructs were generated with the help of the pBluescriptII-SK (Agilent Technologies, Inc., Santa Clara, CA, USA) containing recognition sites for the Tol2 transposase [20]—pBTolmini. 4 × tandem repeats of consensus sites of transcriptions factors were cloned as annealed oligonucleotides with DNA overhang for *Kpn*I (5′) and *Sbf*I (3′), respectively, and cloned into *Kpn*I/*Pst*I-sites of a pBTolmini-E1b-Firefly luciferase SV40polyA vector. The sequences of the individual oligonucleotides can be found in Supplementary Material Table S1.

An analogous approach was used to insert a 2 × tandem repeat of ETS transcription factor binding sites into the same pBTolmini-luciferase backbone. The internal *Xho*I-site introduced by the oligonucleotides 3′ to the 4 × ETS transcription factor consensus sites was used to insert additional oligonucleotides for increasing the number of ETS transcription factor consensus sites to 6 × and 8 × tandems, respectively.

The sequences of the individual ETS binding site containing oligonucleotides can be found in Supplementary Material Table S2.

### 2.2. Cell Culture

PC12 cells (obtained from Division of Cellular Neurobiology, Zoological Institute, TU Braunschweig, Germany, originally derived from RIKEN BRC) were grown in Gibco^TM^ RPMI-1640 (Fisher Scientific, Hampton, NH, USA) medium containing 10% horse serum (Capricorn^TM^ Scientific GmbH, Ebsdorfergrund, Germany) and 5% fetal calf serum (Capricorn). 1321N1 cells (obtained from Sigma-Aldrich, acc. No. 86030402) were cultivated in Gibco DMEM (Fisher Scientific, Inc., Waltham, MA, USA) medium containing 10% fetal calf serum (Capricorn). All media were supplemented with penicillin (0.15 M), streptomycin (86 µM), and glutamine (2 mM).

For induced medium conditioning, 1321N1 cells were plated in a 6-well plate at a density of 1 × 10^5^ cells per well and incubated at 37 °C with 5% CO_2_. After 24 h the media were replaced by serum-reduced medium (Gibco DMEM with 1% fetal calf serum (Capricorn)). After another 24 h incubation period erinacine C, or 0.5% EtOH as solvent control were added and cells were incubated for 48 h before the conditioned supernatants were harvested.

PC12 cells were seeded into collagen coated (0.005%, Hoffmann-La Roche, Basel, Switzerland) 24-well plates at a density of 3 × 10^4^ cells per well. For microscopy analysis collagen coated coverslips were added to the wells. After 24 h of incubation, the medium was replaced by 1321N1 cell conditioned supernatant and the PC12 cells were allowed to differentiate for 48 h at 37 °C with 5% CO_2_. As positive control, serum-reduced medium supplemented with human recombinant NGF (200 ng/mL, SRP3015, Sigma-Aldrich, Inc., St. Louis, MO, USA) was used instead of conditioned supernatant.

### 2.3. Pharmacological Studies

For pharmacological treatments 1321N1 cells were plated in a 6-well plate at a density of 1 × 10^5^ cells per well and incubated at 37 °C with 5% CO_2_. Compounds (PD98059: 20 µM, Sigma-Aldrich, P215; U0126: 10 nM, Sigma-Aldrich, U120; dissolved in DMSO) were diluted in medium to the final concentration containing 1% DMSO and added to the cultured cells for 48 h (controls contained 1% DMSO as well).

Similarly, PC12 cells were seeded into collagen coated (0.005%, Hoffmann-La Roche) 24-well plates at a density of 3 × 10^4^ cells per well. After 24 h of incubation, the serum was replaced by serum-reduced medium containing medium-diluted pharmacological compounds (K252a: 300 nM, Sigma-Aldrich, 05288; PD98059: 20 µM, Sigma-Aldrich, P215; U0126: 10 nM, Sigma-Aldrich, U120; Bisindolmaleimide: 6 µM, Merck KGaA, Darmstadt, Germany, 203290; LY294002: 50 µM, Sigma-Aldrich, L9908). Then, 6 h later either recombinant NGF (200 ng/mL, SRP3015, Sigma-Aldrich) or medium conditioned by 1321N1 cells and supplemented with the same concentration of the compounds was added.

### 2.4. PC12 Cell Differentiation Analysis

PC12 differentiation: PC12 cells were analyzed by transmitted light (DM5500, Leica Microsystems, Wetzlar, Germany) or laser scanning confocal microscopy (SP8, Leica Microsystems) for neurite outgrowth. Cells containing neurites longer than one cell diameter in length were classified as differentiated. Three independent experiments were performed in doublets with 100 randomly picked cells analyzed for each well (*n* = 600 cells analyzed in total) to determine an average differentiation ratio.

Neurite length analysis: in addition, 10 neurites each from six independent randomly selected visual fields recorded by transmitted light microscopy obtained from three independent experiments (180 neurites in total) were measured to determine an average neurite length.

### 2.5. Transfection

PC12 cells were cultured as described above and transfected with plasmids using Fugene HD (Promega, Inc., Fitchbrug, WI, USA) for 48 h according to the manufacturer’s protocol.

To transfect 1321N1 cells the transfection agent (Fugene HD, Promega Inc.) and expression vectors were mixed according to the manufacturer’s instructions. Then, 10 µL of transfection mixture was added to each well of a 96-well plate. Subsequently, 2 × 10^4^ 1321N1 cells were added in a volume of 100 µL followed by incubation for 48 h.

For electroporation 1 × 10^6^ cells were dissolved in 500 µL PBS (Thermo Fisher Scientific), supplemented with 10 µg of plasmid DNA and transferred into an electroporation cuvette (0.4 cm, VWR International, Inc., Radnor, PA, USA, 732-2924). Electroporation occurred with the following conditions: one-time pulse, exponential decay, 300 V, 950 μF (Gene pulser Xcell, BioRad, Inc., Hercules, CA, USA). Electroporated cells were diluted in 500 µL of fresh culture medium. Subsequently, 200 µL of the cell suspension each were plated into a 6-well plate containing 1.8 mL of culture medium and incubated for 24 h at 37 °C with 5% CO_2_.

### 2.6. Luciferase Assays

To quantify transcriptional activity luciferase assays were performed on vector-transfected cells. In general, Firefly luciferase expression vectors were cotransfected with a Renilla luciferase expression plasmid in ratio of 3:1 to adjust obtained data for differences in transfection efficiency. Following incubation, cells were washed in PBS and lysed in 1 × passive lysis buffer (PJK Biotech GmbH, Kleinblittersdorf, Germany) for 15 min at room temperature. Subsequently, the enzymatic activity of Firefly and Renilla luciferase was measured by bioluminescence on a microplate reader (Spark20M, Tecan Ltd., Männedorf, Switzerland) using Beetle Juice (PJK Biotech GmbH) and Renilla juice (PJK Biotech GmbH) as enzymatic substrates, respectively, according to the manufacturer’s protocol. In every experiment, cells transfected with a Firefly luciferase expression construct driven by yeast Gal4 binding sites were used as negative control, of which the measured activity was deducted from all other measured values as background activity. Activity values measured for Renilla luciferase were set as 1. Results are displayed as fold upregulation compared to 0.5% EtOH solvent controls.

### 2.7. Semiquantitative RT-PCR

For semiquantitative reverse transcriptase PCR assays total RNA was isolated from 1321N1 cells using RNAPure™, peqGOLD (PEQLAB Biotechnologie GmbH, Erlangen, Germany). First-strand cDNA synthesis was performed from 2 μg of total RNA using AMV reverse transcriptase (Promega, Inc.) and oligo (dT) primer (Promega, Inc.). The following PCR primers were used for amplifying specific cDNA fragments: *gapdh* (sense: 5′-TCCACCACCCTGTTGCTGTA-3′; antisense: 5′-CCACAGTCCATGCCATCAC-3′; 451 bp), *ngf* (sense: 5′-CCAAGGGAGCAGTTTCTATCCTGG-3′; antisense: 5′-GCAGTTGTCAAGGGAATGCTGAAGTT-3′; 189 bp), *bdnf* (sense: 5′-TAACGGCGGCAGACAAAAAGA-3′; anti-sense: 5′-GAAGTATTGCTTCAGTTGGCCT-3′; 101 bp). The PCR reactions were performed in a 25 μL volume containing cDNA template (2 μL), dNTP (10 mM, 0.5 μL), primers (100 μM; 0.1 μL), Go Taq buffer (5×, 5 μL), and Go Taq polymerase (5 U/μL; 0.2 μL, Promega, Inc.). The amplification programs were started with a step of 94 °C for 2 min and finished by a 72 °C step for 5 min, while following cycles of amplification were used: *gapdh*, 20 cycles: 94 °C, 30″, 60 °C, 30″, 72 °C, 45″; *ngf*, 30 cycles: 94 °C, 30″, 61 °C, 30″, 72 °C, 30″; *bdnf*, 30 cycles: 94 °C, 30″, 58 °C, 30″, 72 °C, 30″. The amplified cDNAs were analyzed by gel electrophoresis on a 1% or 2% agarose gel, respectively, followed by cDNA amount quantification using the open source FIJI imaging analysis software (https://imagej.net/Fiji). Amounts of *gapdh* amplicons were used as reference and set as 1.

### 2.8. Origin of Erinacine C

Erinacine C was isolated from the mycelial culture of *Hericium erinaceus* (strain STMA 06157B) as described previously [7].

### 2.9. Statistical Analysis

For statistical analysis the software Prism v8 (Graphpad Software Inc., San Diego, CA, USA) was used. Initially, the normality of distribution of datasets was tested using Shapiro Wilk and Kolmogorow–Smirnov tests respectively (Tables S2–S14). Subsequently, the significance of difference between the control and experimental groups was determined by one-way ANOVA with post hoc Bonferroni analysis for normally distributed datasets and with Kruksal Wallis analysis for non-normally distributed datasets (*p*-values are shown in Tables S15–S27). Data are displayed as the average ± SEM for the indicated number of independent experiments.

Graphs displaying all individual data points determining the median, minimal, and maximal values are shown in the Supplemental Material Figures S1–S9.

## 3. Results

### 3.1. Erinacine C Induces Expression of Nerve Growth Factor (NGF) in Astrocytoma Cells

**Figure 1:**
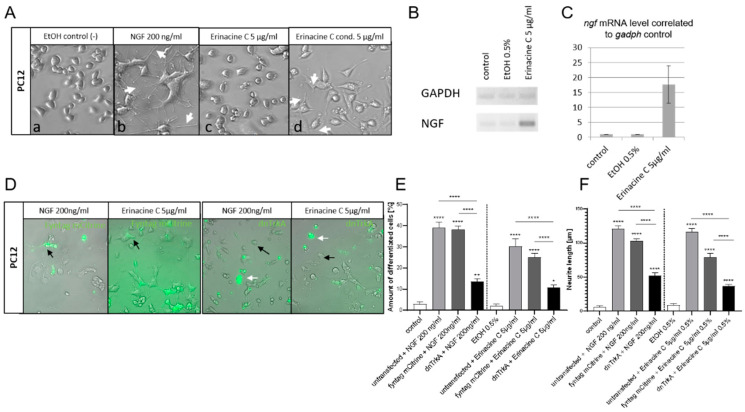
Erinacine C induces NGF expression in 1321N1 astrocytoma cells. (**A**) PC12 cells after 48 h of incubation in medium supplemented with (**a**) 0.5% EtOH, (**b**) 200 ng/mL of recombinant NGF, (**c**) 5 µg/mL erinacine C, (**d**) or maintained in medium conditioned by 1321N1 cells that were incubated with 5 µg/mL erinacine C. Neurite-like membrane protrusions as signs of PC12 differentiation are marked by white arrows. (**B**) PCR fragments after gel electrophoresis of semiquantitative RT-PCR samples to compare regulation of *ngf* expression in 1321N1 cells to the housekeeping gene *gapdh* under normal, 0.5% EtOH, and 5 µg/mL erinacine C conditions 48 h after onset of compound treatment. (**C**) Relative expression levels of *ngf* 1312N1 cells, EtOH controls and erinacine C exposed cells (*n* = 3). (**D**) PC12 cells transfected with pCS-dnTrkA-Citrine (green fluorescence) treated with 0.5% EtOH, 200 ng/mL recombinant NGF or conditioned medium of 1321N1 cells incubated in 5 µg/mL erinacine C. While nonfluorescent and pCS-FynmCitrine transfected control cells are marked by black arrows, green fluorescent cells with impaired TrkA-signaling are marked by white arrows. The differentiation of PC12 cells was quantified regarding (**E**) the percentage of cells displaying membrane protrusions (*n* = 3 independent cultures) and (**F**) the average length of neurite-like structures established by differentiating cells (*n* = 180). * *p* < 0.05, ** *p* < 0.01, **** *p* < 0.0001. Scale bar: 100 µm.

The signaling activity of NGF on cellular behavior can be easily tested with the help of the PC12 cell line [21]. These cells express the high affinity NGF receptor TrkA and respond upon NGF-treatment with a neuronal cell-like differentiation [22]. Therefore, we first validated our PC12 cells by supplementing their media with 200 ng/mL of recombinant NGF. After 48 h of incubation the outgrowth of many neurite-like dendrite protrusions indicating PC12 differentiation could be observed (Figure 1(Ab) white arrows), in contrast to Ethanol (EtOH)-only treated controls, in which PC12 cells remained in a round morphology of undifferentiated progenitor cells (Figure 1(Aa)). Incubation of PC12 cells with a nontoxic concentration of erinacine C of 5 µg/mL did not result in PC12 differentiation (Figure 1(Ac)). Instead treatment of cultured cells of the astrocytoma cell line 1321N1 with erinacine C for 48 h and transferring this conditioned medium to PC12 cultures triggered clear PC12 differentiation indicated by the numerous neuritic structures visible 48 h after medium exchange (Figure 1(Ad) white arrows).

Semiquantitative reverse transcriptase polymerase chain reaction (RT-PCR) analysis revealed that compared to untreated and EtOH controls, erinacine C incubated 1321N1 cells (5 µg/mL) displayed a significant upregulation of *ngfβ* expression compared to the expression of the housekeeping gene *glyceraldehyde 3-phosphate dehydrogenase* (*gapdh*) 48 h after erinacine C exposure (Figure 1B) mounting to a 17-fold upregulation (Figure 1C). These results confirmed that erinacine C does not induce autocrine NGF expression and secretion in PC12 cells directly, but rather activates *ngf* expression in astrocytic cells that could act on neurons in a paracrine manner.

To investigate whether erinacine C induced neurotrophic activity in the 1321N1 conditioned medium mediates PC12 differentiation and neurite outgrowth via the TrkA receptor, we generated a dominant negative TrkA variant by replacing the C-terminal intracellular tyrosine kinase domain with the yellow fluorescent protein Citrine containing an endoplasmatic reticulum (ER) exit signal at its C-terminus. This pCS2+ dnTrkA-Citrine expression vector was transfected into PC12 cells, which were subsequently treated either with NGF-supplemented (200 ng/mL) or erinacine C conditioned medium (5 µg/mL). A pCS2+ vector expressing a membrane targeted mCitrine fluorescent protein (FynmCitrine) served as control. Nontransfected cells displayed under both conditions clear signs of differentiation (Figure 1D black arrows) as did the transfection controls expressing FynmCitrine (not shown). While NGF triggered differentiation of 38.9 ± 2.7% of the cultured PC12 cells (Figure 1E) containing an average length of outgrown neurites of 120.8 ± 4.5 µm (Figure 1F), erinacine C conditioned 1321N1 medium yielded a percentage of 30.3 ± 3.3% differentiated PC12 cells (Figure 1E) with neurites of 116.2 ± 5.2 µm in length (Figure 1F). Cells transfected with FynmCitrine behaved similarly leading upon NGF-treatment to an induction of differentiation of 38.2 ± 1.8% of fluorescent PC12 cells (Figure 1E) with an average neurite length of 102.9 ± 3.1 µm. Incubation with erinacine C conditioned 1321N1 medium resulted in differentiation of 25.2 ± 1.7% of transfected PC12 cells with an average neurite length of 79.1 ± 5.3 µm. In contrast fluorescent cells with inhibited TrkA signaling remained largely undifferentiated and resembled nontreated or EtOH-only controls. Despite stimulation with recombinant NGF, only 13.5 ± 1.3% of the cultured fluorescent PC12 cells displayed signs of differentiation (Figure 1E) with significantly shorter neurites of 51.8 ± 4.5 µm in length (Figure 1F); analogous to fluorescent TrkA-inhibited PC12 cells cultured in erinacine C conditioned 1321N1 medium of which only 10.9 ± 1.0% of the PC12 cells differentiated (Figure 1E) with short neurites of 36.3 ± 2.6 µm length on average (Figure 1F). This residual but significant amount of PC12 differentiation compared to controls argues for a residual TrkA activity in the presence of the dominant-negative TrkA variant. Nevertheless, these findings substantiate that erinacine C induces *ngf* expression in 1321N1 astrocytoma cells with NGF accumulating in the supernatant and subsequently eliciting PC12 differentiation via binding and activating TrkA. Although we have previously shown that erinacine C is able to also induce the expression of BDNF in 1321N1 cells, secreted BDNF activity cannot be responsible for inducing PC12 differentiation as these cells lack expression of the corresponding BDNF receptor TrkB [23].

### 3.2. Erinacine C Conditioned Astrocytoma Medium Likely Acts via Known TrkA-Mediated Signaling Pathway

**Figure 2:**
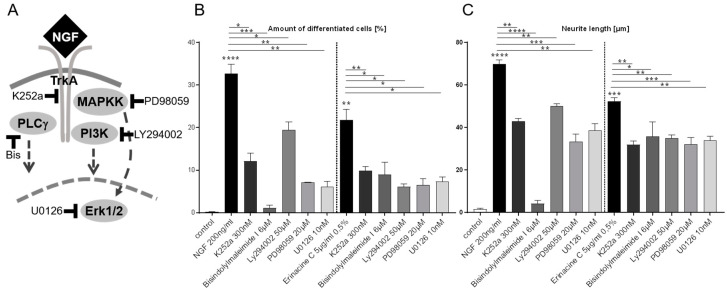
Erinacine C induced neurotrophic activity mediates PC12 differentiation via MAPK/ERK, PLCγ and PI3K signaling. (**A**) Schematic drawing of signaling pathways downstream of the NGF-activated TrkA receptor and respective pharmacological inhibitors. (**B**) Percentage of PC12 cells differentiated into neuron-like cells upon stimulation with either recombinant NGF (left) or conditioned medium of erinacine C treated 1321N1 astrocytoma cells (right) in the presence of signal transduction cascade inhibitors (two independent fields, *n* = 100 cells; three independent cultures). (**C**) In the same cultures individual neurite length of differentiated cells were measured and are displayed as average values (*n* = 180 cells, three independent cultures) as an indication of the extent of differentiation. * *p* < 0.05, ** *p* < 0.01, *** *p* < 0.001, **** *p* < 0.0001.

Extracellular stimulation of the TrkA receptor by NGF results intracellularly in the activation of three major signaling cascades encompassing the mitogen activated protein kinase/extracellular signal-regulated kinase pathway (MAPK/ERK pathway), phosphoinositide-3-kinase pathway (PI3K pathway), and the phospholipase C gamma pathway (PLCγ pathway), all of which are involved in NGF-mediated PC12 differentiation and neurite outgrowth [24] (Figure 2A). To further investigate whether neurotrophic activity in the erinacine C conditioned 1321N1 medium activates these pathways in PC12 cells, we supplemented the conditioned 1321N1 medium with inhibitors selective for each of the different signaling branches downstream of TrkA-activity (Figure 2A).

After incubation of PC12 cells for 48 h, the percentage of differentiated cells containing neurites as well as the average length of extended neurites were quantified. Recombinant NGF (200 ng/mL) triggered the differentiation of 32.7 ± 2.1% of the cultured PC12 cells (Figure 2B), which displayed an average neurite length of 69.8 ± 1.9 µm (Figure 2C). This differentiation was significantly reduced to a differentiation rate of 12.1 ± 1.7% (Figure 2B) and an average neurite length of 42.8 ± 1.3 µm (Figure 2C) in the presence of the TrkA inhibitor K252a. Similarly, erinacine C conditioned 1321N1 medium stimulated differentiation of 21.8 ± 2.5% of the PC12 cells (Figure 2B) displaying an average neurite length of 52.2 ± 1.7 µm (Figure 2C), that was reduced by K252a to 9.9 ± 0.9% differentiation (Figure 2B) and an average neurite length of 31.9 ± 1.6 µm (Figure 2C), respectively. These independent findings compare well to the results obtained with the coexpression of the dominant negative TrkA receptor.

Next, signaling events downstream of TrkA were investigated in an analogous manner using specific pharmacological inhibitors at previously published concentrations [22,25,26,27]. Either stimulation of PC12 cells with recombinant NGF or erinacine C conditioned 1321N1 medium combined with the simultaneous pharmacological inhibition of either PLCγ (6 µM Bisindolylmaleimide I, Bis), PI3K (50 µM LY294002), MAPKK (20 µM PD98059), or ERK1/2 (10 nM U0126) significantly reduced the percentage of differentiated PC12 cells with varying severity (Figure 2B) and decreased to a different extent the average neurite length (Figure 2C). Thus, erinacine C induced neurotrophic activity secreted from 1321N1 astrocytoma cells—like recombinant NGF—mediates the differentiation of PC12 cells by likely activating PLCγ, PI3K, and MAPK a pattern of intracellular signaling characteristic for neurotrophin-stimulated Trk receptors. Yet, a detailed understanding of the signaling events downstream of TrkA-activation by erinacine C conditioned medium will have to await phosphorylation studies on the protein level.

### 3.3. Transcription Factor Mediated Signal Transduction Activated by Erinacine C

**Figure 3:**
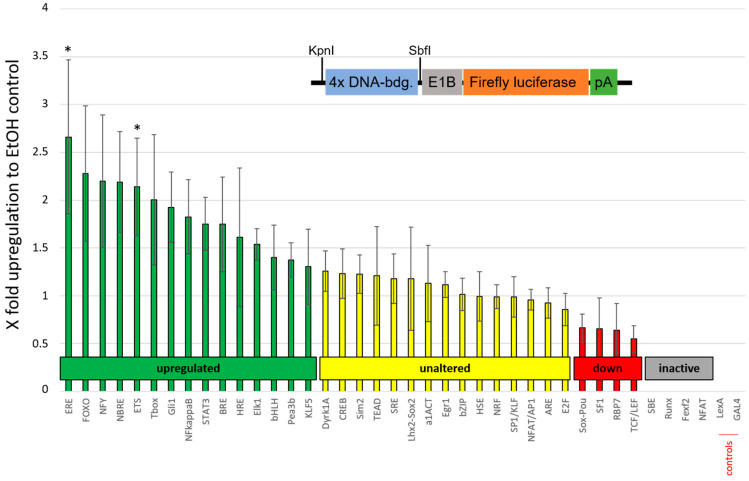
Erinacine C differentially regulates reporter construct expression with binding sites for various transcription factors. Schematic drawing shows organization of Firefly luciferase reporter constructs containing a linear array of four consensus sequeces for transcription factor bindings sites (blue) followed by the weak basal promoter E1b (grey) and the cDNA coding for Firefly luciferase (orange) followed by the SV40 polyadenylation sequence (green). The sequences for the respective binding sites are provided in Table S1. Transcriptional activation of these constructs in 1321N1 cells upon incubation with erinacine C is displayed in relation to 0.5% EtOH solvent controls as fold-activation. Upregulated transcription above 1.3-fold over EtOH controls is shown in green color, while constructs with downregulated transcription below 0.7-fold with respect to EtOH controls are depicted in red color, these values for classifying and up- or downregulation were chosen arbitrarily for facilitating the readability of the data (four values per treatment, four independent cultures). * *p* < 0.05.

Having revealed the intracellular signaling cascades in neural PC12 cells downstream of the TrkA receptor activated by erinacine C conditioned astrocytic medium, we wondered about transcriptional processes activated by erinacine C in astrocytic cells themselves. We therefore constructed a small library of reporter constructs in which Firefly luciferase expression is driven by a repetitive linear organization of four transcription factor consensus binding sites (Figure 3 schematic drawing of reporter gene cassette and Table S28). These regulatory sequences included desoxyribonucleic acid (DNA)-binding transcription factors that are known to act upstream of *ngfβ* expression [14,15]. The constructs were transfected into 1321N1 cells together with a plasmid expressing *Renilla* luciferase under control of the cytomegalovirus (CMV) promoter in the pCS2+ vector backbone [16] to normalize expression data for differences in transfection efficiencies. After 24 h of erinacine C (5 µg/mL) incubation, cells were harvested, lysed, and analyzed for luciferase expression exploiting the conversion of Firefly and *Renilla* luciferase specific bioluminescent substrates that were quantified with the help of a microplate reader. Ratios of Firefly to *Renilla* luminescence were normalized to 0.5% EtOH-treated control samples of 1321N1 cells and were displayed as fold up- or downregulation in comparison to these controls.

Several of these plasmids did not give any signal including constructs with consensus binding sites for the transcription factor Small Mothers Against Decapentaplegic (Smad3/4) (involved in TGF-signaling), Runt related transcription factor (Runx), Forebrain Embryonic Zinc Finger (Fez), and Nuclear Factor of activated T-cells (NFAT) suggesting that the respective pathways are inactive in 1321N1 astrocytic cells. This also included constructs with consensus bindings sites for the yeast regulatory protein Gal4 and bacterial LexA transcription factors, which served as additional controls (Figure 3, construct activity marked in grey). Reporter constructs resulting in a drop of luciferase activity below 0.7 upon erinacine C treatment compared to the respective EtOH control were considered as downregulated signal transduction pathways with their respective average values marked in red color (Figure 3). Plasmids leading to an increase of luciferase activity above 1.3-fold upon erinacine C treatment were classified as upregulated signaling cascades with their individual average fold-activity displayed in green color (Figure 3). Both values for classifying pathways as up- or downregulated were chosen arbitrarily and are not based on an actual reference value. Here several reporter constructs reflecting particular signaling pathways yielded an upregulation of expression involving transcriptional activation for example by Forkhead Box Proteins (FOXO), Glioma-associated oncogene (Gli) or Polyomavirus enhancer activator (Pea) transcription factors (Figure 3 see columns labeled in green). In particular, reporter constructs containing consensus binding sites for estrogen receptors (ERE) and E26 transformation specific (ETS) displayed an upregulation of the luciferase reporter that was statistically significant.

### 3.4. Erinacine C Activates ETS-Dependent Transcription

**Figure 4:**
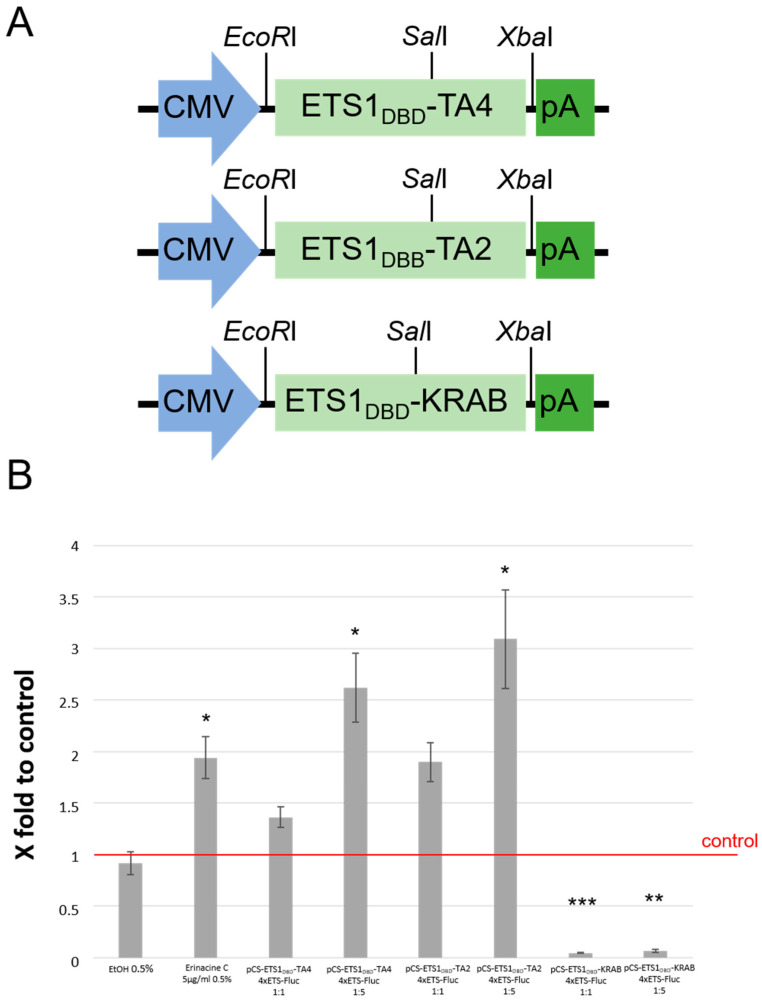
Validation of ETS reporter construct. (**A**) Schematic drawings of pCS2+ expression vectors with cDNAs of fusions between the human ETS1 DNA binding domain and either transcriptional activation (TA4, TA2) or repression (KRAB) domains. These were transfected into 1321N1 cells together with the ETS Firefly luciferase reporter construct (see Figure 3). (**B**) Fold activation of 4 x ETS Firefly luciferase reporter in the presence of ETS1 transcriptional activator and repressor constructs transfected at equal amounts are at five-fold excess. Reporter construct activation alone in 1321N1 cells due to endogenous ETS-activity was set as one (marked as red line) (four values per treatment, four independent cultures). * *p* < 0.05, ** *p* < 0.01, *** *p* < 0.001.

As ETS-, Elk1-, and Pea3 DNA binding sites share a high degree of sequence overlap and belong to the same class of ETS-transcription factors, upregulation of all three transcriptional reporters upon erinacine C treatment serves as independent control for ETS-mediated transcriptional activation by erinacine C. This independent validation of increased ETS-dependent transcriptional activation by erinacine C prompted us to study this activation in further detail. To first support that this reporter construct indeed detects ETS-signaling and to further corroborate the ETS-dependent transcription of Firefly luciferase in the reporter construct driven by four ETS DNA consensus binding sites, we cloned a nuclear localization signal and the DNA binding domain (DBD) of human ETS1 in frame with the TA2, TA4, or KRAB transcriptional activation or repressor domains [17,19], respectively, into the backbone pCS2+ (Figure 4A). Cotransfection of these expression vectors together with the ETS-luciferase reporter construct into 1321N1 cells followed by luciferase activity assays 24 h after transfection revealed that indeed Firefly luciferase activity was upregulated upon coexpression with the ETS transcriptional activators ETS1_DBD_-TA2 and ETS1_DBD_-TA4, but silenced upon coexpression with the ETS transcriptional repressor ETS1_DBD_-KRAB in a concentration dependent manner (Figure 4B). This confirms that the ETS luciferase reporter construct activated by erinacine C treatment of 1321N1 astrocytic cells indeed responds to altered ETS1-activity. Yet, in particular for the repressor variant ETS1_DBD_-KRAB it has to be kept in mind ETS signaling with homo-and heterodimerization of ETS transcription factors is complex and that the activators and repressor established may not mimic or interfere with all types of ETS-mediated transcriptional activation.

**Figure 5:**
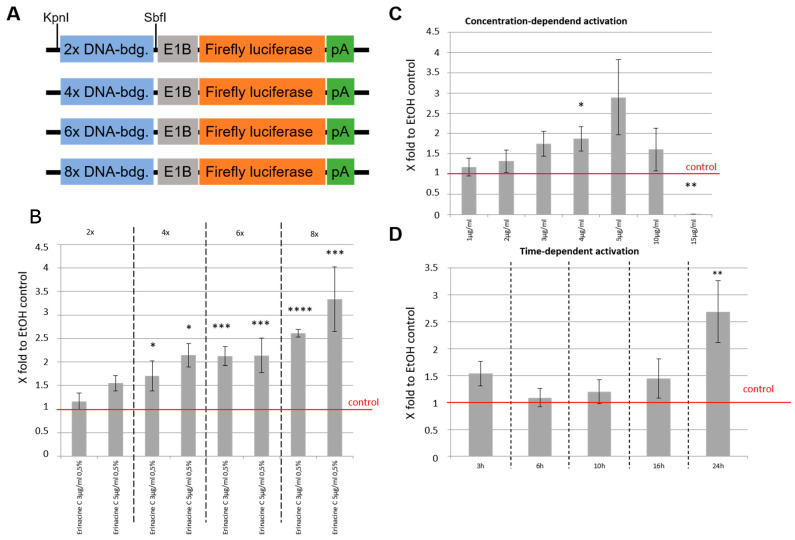
Erinacine C activates ETS-reporter construct in a concentration dependent manner. (**A**) Series of constructed reporter plasmids containing 2, 4, 6, or 8 ETS1 binding sites followed by the E1b minimal promoter and Firefly luciferase. (**B**) Transfection of these constructs into 1321N1 cells followed by incubation with either 3 or 5 µg/mL of erinacine C for 24 h. Fold-upregulation of luciferase activity in dependence of the different erinacine C concentrations and increasing numbers of ETS binding sites are displayed. (**C**) Fold-upregulation of luciferase activity with the 8 x ETS reporter construct upon increasing erinacine C concentrations up to 5 µg/mL. Higher erinacine C concentrations yielded lower luciferase activity values probably due to altered expression responses in 1321N1 cells. (**D**) Fold-upregulation of luciferase activity with the 8 x ETS reporter construct and 5 µg/mL erinacine C incubation in dependence of varying incubation times. EtOH treated cells served as controls for which luciferase activity was set as one in all displayed assays (red line) (four values per treatment, four independent cultures). * *p* < 0.05, ** *p* < 0.01, *** *p* < 0.001, **** *p* < 0.0001.

To investigate whether the ETS reporter responds to altered numbers of ETS bindings sites we used reporter constructs with linear tandems of 2, 4, 6, and 8 ETS DNA binding sites (Figure 5A). These plasmids were transfected into 1321N1 cells followed by stimulation with either 3 or 5 µg/mL erinacine C and Firefly luciferase activity measurements after a 24 h incubation period. Both concentrations yielded a successive increase in luciferase activity with increasing numbers of ETS binding sites (Figure 5B) reaching 2.6 ± 0.1 or 3.3 ± 0.7-fold activation over values of 0.5% EtOH controls (red line).

Furthermore, the reporter construct with eight ETS DNA binding sites was used for 1321N1 transfections and cells were treated with successively increasing concentrations of erinacine C ranging from 1 to 5 µg/mL in steps of 1 µg/mL each. Again fold-activation of Firefly luciferase activity increased steadily with raising erinacine C reaching at a concentration of 5 µg/mL a 2.9 ± 0.9-fold activation over 0.5% EtOH controls as maximum (Figure 5C). Reduced levels of fold-activation of Firefly luciferase at 10 µg/mL erinacine C, a concentration tested to lack cytotoxicity, could reflect that such high concentrations of erinacine C induce the expression of further signal transduction molecules, which attenuate the transcriptional activation from the ETS consensus binding site.

Next, the same experiments were repeated with the 8 ×reporter construct and 5 µg/mL erinacine C, but Firefly luciferase activity was measured at varying time periods of erinacine C incubation ranging from 3 to 24 h. This showed that increased levels of ETS dependent Firefly luciferase activity were obtained after about 10 h of erinacine C treatment (Figure 5D). Firefly luciferase activity increased further with prolonged incubation times reaching significant levels of 2.7 ± 0.6-fold after 24 h of erinacine C treatment.

Therefore, induction of ETS consensus binding site dependent transcriptional activity by erinacine C is concentration dependent in 1321N1 cells with the number of ETS sites acting in an additive manner but requiring about 10 h to respond to erinacine C stimulation.

### 3.5. Erinacine C is Unlikely to be Sufficient to Induce ngf Transcription in 1321N1 Astrocytic Cells

**Figure 6:**
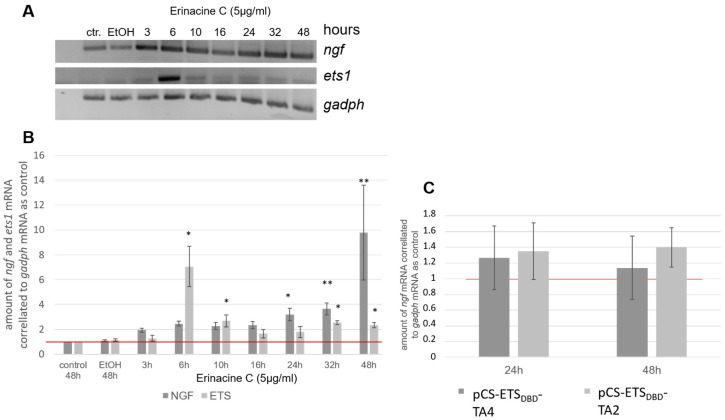
ETS-activity alone may not induce NGF expression in astrocytic 1321N1 cells. (**A**) Semiquantitative RT-PCR against *ets1*, *ngf*, and *gapdh* (control) mRNA expression in erinacine C incubated 1321N1 cells after various timepoints of compound incubation. (**B**) Average ratios of *ets1* or *ngf* compared to *gapdh* mRNA (*n* = 3 independent RT-PCRs) were displayed against different erinacine C incubation periods. As controls untreated (ctr.) or EtOH-treated 1321N1 cells were used. (**C**) *ngf/gapdh* mRNA ratios after transfection of astrocytic 1321N1 cells with the constitutively active transcriptional activators ETS1_DBD_-TA4 or ETS1_DBD_-TA2 followed by 24 and 48 h of incubation, respectively. *ets1*/*gapdh* mRNA ratio in untreated cells incubated for 48 h with serum-reduced medium was set as one indicated by a red line in B and C (three independent cultures). * *p* < 0.05, ** *p* < 0.01.

Erinacine C induced significant ETS consensus binding site dependent Firefly luciferase activity after 24 h of 1321N1 incubation (Figure 5D). This induction likely requires transcription, translation, and nuclear import of ETS transcription factors like ETS1 followed by transcription, translation, and substrate conversion by the luciferase. Increase of erinacine C induced *ets1* mRNA is therefore expected to occur significantly earlier than the observed increase in luciferase activity, which we tested by semiquantitative RT-PCR at various time points of erinacine C (5 µg/mL) incubated 1321N1 cells compared to *gapdh* mRNA levels (Figure 6A). The ratio of mRNA levels of *ets1* mRNA to *gapdh* mRNA in untreated cells served as control and was set as one (Figure 6B, red line). Indeed, *ets1* mRNA levels showed an about 7-fold increase of mRNA levels over *gapdh* mRNA concentrations already after 6 h of erinacine C incubation (Figure 6B) and therefore 18 h before the observation of significant ETS consensus binding site dependent luciferase activity. Interestingly though, *ets1* mRNA levels only peaked for a short time and dropped to a slightly elevated upregulation 4 h later (Figure 6B).

If NGF expression is a direct target of ETS transcriptional activation, we would expect a time dependency of *ngf* mRNA elevation, with upregulation of *ngf* mRNA to occur after *ets1* mRNA upregulation, but before significant ETS-dependent luciferase activity observed at 24 h of erinacine C incubation. However, semiquantitative RT-PCR revealed a slight but steady increase of *ngf* mRNA starting at around 24 h after onset of erinacine C treatment and further increased until 32 h to 3.6 ± 0.5. At 48 h a strong induction to almost 10-fold (9.8 ± 3.8) levels compared to *gapdh* mRNA could be observed. These findings argue against *ngf* expression being a direct target of ETS transcriptional activation.

To test if ETS-dependent transcriptional activation is sufficient to induce *ngf* mRNA expression semiquantitative RT-PCR against *ngf* compared to *gapdh* mRNA levels were repeated in 1321N1 cells that had been transfected with the constitutive ETS1_DBD_-TA4 and ETS1_DBD_-TA2 transcriptional activators (Figure 4A). Neither 24 nor 48 h after transfection of these transcriptional activators a significant upregulation of *ngf* mRNA levels could be observed (Figure 6C) suggesting that activation of ETS activity alone by erinacine C is not sufficient to induce *ngf* expression in 1321N1 cells. It has to be kept in mind though that the established ETS-activator variants ETS1_DBD_-TA4 and ETS1_DBD_-TA2 may not mimic all kinds of ETS factor mediated transcriptional activation.

### 3.6. Erinacine C Induced ETS Activity May Occur Independently of ngf and bdnf Induction

**Figure 7:**
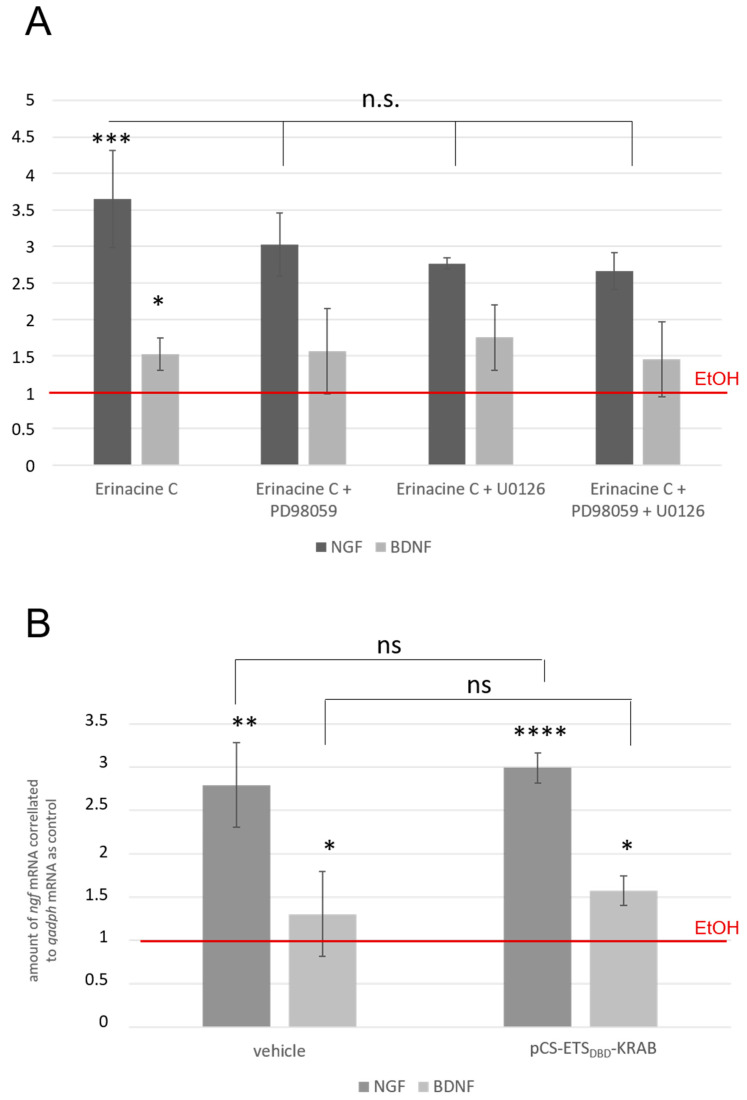
Erinacine C likely activates ETS signaling independent of inducing neurotrophin expression. (**A**) Fold-activation of *ngf* (dark grey) and *bdnf* (light grey) transcription in 1321N1 cells treated for 48 h with erinacine C in the presence of MAPKK (20 µM PD98059) and ERK1/2 (10 nM U0126) inhibitors compared to *gapdh* mRNA analyzed by semiquantitative RT-PCR. Values from 0.5% EtOH treated cells were used as control and were set as one (red line). Stars indicate a significant upregulation compared to EtOH controls. (**B**) Fold-activation of *ngf* (dark grey) and *bdnf* (light grey) expression by semiquantitative RT-PCR from 1321N1 cells electroporated either with the pBluescriptII vector (vehicle) or the pCS-ETS1_DBD_-KRAB construct (KRAB) expressing a constitutive inhibitor of ETS signaling. A total of 24 h after transfection cells were exposed to erinacine C (5 µg/mL) or EtOH (0.5%) for 48 h. The latter served as control with its *ngf*:*gapdh* ratio set as one (marked as red line) (three independent cultures). ns: not significant; * *p* < 0.05, ** *p* < 0.01, *** *p* < 0.001, **** *p* < 0.0001.

To clarify whether ETS activity is required to induce *ngf* mRNA expression, we first made use of pharmacological inhibitors. ETS signaling is usually activated via the MAPKK-ERK1/2 pathway [28]. We therefore incubated 1321N1 cells with 5 µg/mL erinacine C together with either 20 µM PD98059 (MAPKK inhibitor) or 10 nM U0126 (ERK1/2 inhibitor) for 48 h followed by total RNA isolation, cDNA preparation, and semiquantitative RT-PCR to determine the ratio of *ngf* mRNA levels compared to *gapdh* mRNA. This ratio obtained from 0.5% EtOH treated cells was set as one (Figure 7A, red line). First, induction of *ngf* mRNA by erinacine C treatment was confirmed as positive control reaching levels of 3.7 ± 0.7-fold upregulation compared to EtOH-only treated control cells. Addition of either pharmacological inhibitor or adding both of them in combination resulted in a small but nonsignificant shift to lower values of erinacine C mediated *ngf* mRNA upregulation. Yet, this result remained ambiguous as the upregulation of *ngf* transcription in the presence of the inhibitors was still observable but statistically not significant anymore (Figure 7A). Interestingly the same observations were made for erinacine C mediated expression of *bdnf* mRNA.

To further test the involvement of activated ETS signaling directly in erinacine C induced *ngf* and *bdnf* expression, we electroporated the constitutive ETS transcriptional repressor ETS1_DBD_-KRAB into 1321N1 cells. This transfection method reaches up to 85% transfection efficiency in 1312N1 cells. Electroporation of the pBluescriptII vector instead of the repressor served as control (Figure 7B, vehicle). A total of 24 h after transfection, cells were exposed to erinacine C (5 µg/mL) and incubated for up to 48 h. Subsequently, semiquantitative RT-PCR was performed against *ngf, bdnf*, and *gapdh* mRNA respectively, while values obtained from EtOH-treated cells served as reference and were set as one (Figure 7B). Although around 15% of the 1321N1 cells were not transfected, a significant drop in *ngf* mRNA induction would be expected, if erinacine C induced upregulation of *ngf* or *bdnf* expression were mediated via ETS activity. Yet, neither a reduction in *ngf* nor in *bdnf* mRNA expression in the presence of the ETS1_DBD_-KRAB repressor could be observed. This suggests that erinacine C induces ETS activity in astrocytic 1321N1 cells independently of activating neurotrophin transcription. Alternatively, the established ETS1_DBD_-KRAB inhibitor does not impair ETS signaling events specific for erinacine C mediated upregulation of *ngf* mRNA. Identification of the particular ETS transcription factors induced by erinacine C and their specific inhibition promises to reveal an answer to this interesting question.

## 4. Discussion

In the past years, extracts and isolated secondary metabolites from mycelial cultures and basidiomes of the edible mushroom *Hericium erinaceus* have attracted much attention in the field of chemical biology and pharmacology, because of their broad activity spectrum in supporting the maintenance and restoration of health as well as in counteracting acute and chronic pathogenic disease processes [1,8,9,10,13,22]. In particular for the central nervous system, this medicinal activity is ascribed to the potential of selected cyathane diterpenoids named erinacines to induce the expression of neurotrophins. These small secreted and diffusible ligands support neuronal survival and homeostasis, regulate neuronal plasticity, and promote neuronal regeneration [29,30,31]. We have recently identified erinacine C as potent inducer of the neurotrophin nerve growth factor NGF in cultured astrocytic cells [7].

Here, we revealed that culture medium conditioned by erinacine C treated astrocytic cells induced the differentiation of PC12 cells into neural-like cells indicated by the extension of membrane protrusions reminiscent of neurites. This differentiation could be impaired for the most part by inhibiting TrkA receptor signaling suggesting that the PC12 differentiation activity of the erinacine C conditioned medium is mediated by glial-derived secreted NGF. Interference with TrkA signaling did not entirely suppress PC12 differentiation to the level of PC12 cells treated with media from solvent control incubated astroglial cells. Yet, when NGF-supplemented media was used, a similar reduction but not complete loss of differentiation of PC12 cells with impaired TrkA signaling was observed. This suggests that expression of the dominant negative TrkA receptor variant diminishes, but does not completely disrupt TrkA signal transduction. Still, results from PC12 differentiation and neurite outgrowth are somewhat heterogenous when comparing PC12 cells treated with recombinant NGF versus cells incubated in conditioned medium of erinacine C treated 1321N1 cells. This could be explained by erinacine C inducing lower amounts of NGF compared to the amounts of recombinant NGF protein added as positive control. Moreover, erinacine C induces a number of transcriptional responses from various transcription factor consensus binding sites (Figure 3) suggesting that erinacine C may induce the expression of additional secreted factors besides NGF, and this cocktail of factors in the conditioned medium is less well defined that could elicit variations in the differentiation response of PC12 cells. Downstream of TrkA, conditioned media of erinacine C treated astroglial cells when supplemented with pharmacological inhibitors against the PLCγ, PI3K, and MAPK/ERK pathways was impaired to mediate PC12 differentiation, suggesting an involvement of these three major branches of TrkA signaling in mediating the response to the conditioned medium.

While the *ngf*-inducing properties of erinacine C were confirmed, the effects that erinacine C exerts on astrocytic cells directly have remained elusive. Likely, erinacine C influences the expression in these astrocytic cells on the transcriptional level probably including also factors that regulate *ngf* expression. As a first step we focused on transcriptional processes of selected transcriptional activators with known DNA consensus binding sites that are altered in their activity upon incubating 1321N1 astrocytic cells with erinacine C. This approach should provide access to genetic tools for further quantifying erinacine C activity. A number of the 38 tested consensus DNA binding sites of transcription factors were affected in their ability to drive reporter gene expression upon erinacine C exposure of 1321N1 cells. These included transcriptional activation of reporter constructs by estrogen receptors (ER), Signal Transducer and Activator of Transcription 3 (STAT3), Forkhead Box Protein O (FoxO), or Glioma associated Oncogene family zinc finger 1 (Gli1) transcription factors among others. Activation of c-jun/c-fos-signaling as observed for erinacine A [25] could not be confirmed for erinacine C treated 1321N1 cells. These findings propose that erinacine C appears to exert a rather pleiotropic transcriptional response in astrocytic cells. Yet, the current studies do not distinguish between transcriptional responses induced directly by erinacine C and indirect ones activated further downstream for example in response to ETS-signaling. Real-time RT-PCR could help to resolve temporal differences in transcriptional activation. The activation of *ets1* transcription upregulated already 6 h after erinacine C stimulation argues for an early, probably direct, effect of erinacine C on *ets1*.

Further luciferase assay analysis revealed that the transcriptional activity of ETS consensus binding transcriptional activators responded to erinacine C in a dose dependent manner in 1321N1 cells starting to rise from about 10 to 24 h after compound administration. This corresponds to an observed upregulation of *ets1* mRNA about 6 h after erinacine C administration. Yet, ETS activity mimicked by transcriptional activators containing the ETS1 DNA binding domain failed to upregulate *ngf* transcription, while an analogous repressor was not able to suppress *ngf* transcription. While the possibility exists that these activating and inhibiting ETS variants do not mediate or interfere with all ETS signaling events to their full extent, these data suggest that induction of ETS transcriptional activity by erinacine C represents an additional response of 1321N1 cells to erinacine C treatment independent of increasing NGF secretion. This finding is not surprising considering the many different cellular effects ascribed to erinacines, making it unlikely that all of them are mediated by NGF alone. However, these data provide a first insight into the transcriptional alterations of prominent signal transduction pathways in 1321N1 astrocytic cells induced by the powerful erinacine C compound.

Moreover, these results are exciting in the light of recently published data regarding the involvement of ETS transcription factors in neural cell proliferation during both embryonic development and adult neurogenesis as well as regeneration in various brain regions [32,33]. Induction of ETS activity independent of and in addition to inducing neurotrophin expression could be a first step into understanding a prominent role of erinacines in regulating neurogenesis. Such an activity will broaden the current narrow focus of erinacine compound research in modulating neurotrophin availability. ETS-mediated activation of neurogenesis could well contribute to amelioration of neurodegeneration as has been demonstrated for erinacines in animal models of Alzheimer’s disease [9,10,11]. Therefore, further identifying transcription factors upstream of erinacine C mediated neurotrophin induction as well as characterizing the implications of erinacine C activated transcription factors represents a valuable research approach towards clarifying the mechanism of action of this cyathane diterpenoid. Due to the promising medicinal activities of erinacines combined with their bioavailability and good cellular tolerance these compounds deserve further mechanistic studies.

## 5. Conclusions

In summary, we confirmed that the recently discovered cyathane diterpenoid erinacine C induces expression of the neurotrophins NGF and BDNF in glial cells, and we revealed potential signaling cascades downstream of NGF mediating differentiation in neural-like PC12 cells. In addition, we demonstrated that likely independently of NGF induction, erinacine C promotes ETS-dependent transcription in astroglial cells, which could well play a role in regulating proliferation and regeneration in the central nervous system—a function previously ascribed to *Hericium erinaceus* extracts. Furthermore, having focused on transcriptional targets activated by erinacine C in glial cells should provide entry points for establishing genetic sensors for erinacine C activity. This knowledge could be used for further revealing the mechanism of action as well as for substantiating the physiological properties of this neuromodulatory fungal secondary metabolite in vivo.

## Supplementary Materials

The following are available online at https://www.mdpi.com/2218-273X/10/10/1440/s1, Tables S1–S27: Statistical analysis of luciferase assays, Table S28: List of Firefly luciferase constructs containing four transcription factor DNA binding sites, Table S29: List of Firefly luciferase constructs containing 2–8 transcription factor DNA binding sites, Figures S1–S9: Scatter plots displaying individual data points used for statistical analysis.
