# Supplementary material for: Erinacine C Activates Transcription from a Consensus ETS DNA Binding Site in Astrocytic Cells in Addition to NGF Induction
Source: Biomolecules. 2020 Oct 14;10(10):1440. doi: 10.3390/biom10101440 (PMC7602259; doi:10.3390/biom10101440)
Supplement: Supplementary file 1 [file biomolecules-10-01440-s001.pdf]

## Supplements:

**Table S1:** Sample sizes. The amount of samples is shown for each experiment. Firstly, each single experiment has an amount of measured values, the experiments were done in three to four independent single experiments.

| Experiment | Per sample | Amount of independent experiments | Whole data set |
|---|---|---|---|
| Figure 1A | 2 independent fields with 100 cells | 3 | 6 fields with 600 cells |
| Figure 1B and C | 1 RNA isolation per treatment | 3 | 3 |
| Figure 1D + E | 2 independent fields with 100 cells | 3 | 6 fields with 600 cells |
| Figure 1F | 6 x 10 neurites | 3 | 180 |
| Figure 2B | 2 independent fields with 100 cells | 3 | 6 fields with 600 cells |
| Figure 2C | 6 x 10 neurites | 3 | 180 |
| Figure 3 | 4 values per treatment | 4 | 16 |
| Figure 4B | 4 values per treatment | 4 | 16 |
| Figure 5B + C + D | 4 values per treatment | 4 | 16 |
| Figure 6A + B + C | 1 RNA isolation per treatment | 3 | 3 |
| Figure 7A +B | 1 RNA isolation per treatment | 3 | 3 |

**Table S2:** Test of normal distribution of the values shown in Figure 1E. Normal distribution was tested with Shapiro-Wilk test and Kolmogrov-Smirnov, the p-values were shown (ns not significant p > 0.05; * p < 0.05; ** p < 0.01).

| p-values | Shapiro-Wilk test | Normal distribution | Significance | Kolmogorov-Smirnov | Normal distribution | Significance |
|---|---|---|---|---|---|---|
| **control** | <0.0001 | no | **** | <0.0001 | no | **** |
| **untransfected + NGF 200ng/ml** | 0.9069 | yes | ns | >0.1000 | yes | ns |
| **Fyntag mCitrine + NGF 200ng/ml** | 0.5527 | yes | ns | >0.1000 | yes | ns |
| **dnTrkA + NGF 200ng/ml** | 0.1991 | yes | ns | 0.0888 | yes | ns |
| **EtOH 0.5%** | <0.0001 | no | **** | <0.0001 | no | **** |
| **Untransfected + Erinacine C 5µg/ml 0.5%** | 0.7581 | yes | ns | >0.1000 | yes | ns |
| **Fyntag mCitrine + Erinacine C 5µg/ml 0.5%** | 0.0021 | no | ** | 0.0778 | yes | ns |
| **dnTrkA + Erinacine C 5µg/ml 0.5%** | 0.0110 | no | * | >0.1000 | yes | ns |

**Table S3:** Test of normal distribution of the values shown in Figure 1F. Normal distribution was tested with Shapiro-Wilk test and Kolmogrov-Smirnov, the p-values were shown (**** p < 0.0001).

| p-values | Shapiro-Wilk test | Normal distribution | Significance | Kolmogorov-Smirnov | Normal distribution | Significance |
|---|---|---|---|---|---|---|
| **control** | <0.0001 | no | **** | <0.0001 | no | **** |
| **untransfected + NGF 200ng/ml** | <0.0001 | no | **** | 0.0166 | no | * |
| **Fyntag mCitrine + NGF 200ng/ml** | <0.0001 | no | **** | <0.0001 | no | **** |
| **dnTrkA + NGF 200ng/ml** | <0.0001 | no | **** | <0.0001 | no | **** |
| **EtOH 0.5%** | <0.0001 | no | **** | <0.0001 | no | **** |
| **Untransfected + Erinacine C 5µg/ml 0.5%** | <0.0001 | no | **** | 0.0147 | no | * |
| **Fyntag mCitrine + Erinacine C 5µg/ml 0.5%** | <0.0001 | no | **** | <0.0001 | no | **** |
| **dnTrkA + Erinacine C 5µg/ml 0.5%** | <0.0001 | no | **** | <0.0001 | no | **** |

**Table S4:** Test of normal distribution of the values shown in Figure 2B. Normal distribution was tested with Shapiro-Wilk test and Kolmogrov-Smirnov, the p-values were shown (ns not significant p > 0.05; * p < 0.05; ** p < 0.01; *** p < 0.001; **** p < 0.0001).

| p-values | Shapiro-Wilk test | Normal distribution | Significance | Kolmogorov-Smirnov | Normal distribution | Significance |
|---|---|---|---|---|---|---|
| **Control** | <0.0001 | no | **** | <0.0001 | no | **** |
| **NGF 200ng/ml** | 0.2832 | yes | ns | >0.1000 | yes | ns |
| **K252a 300nM** | 0.2274 | yes | ns | >0.1000 | yes | ns |
| **Bisindolyl-maleimide I 6µM** | 0.8998 | yes | ns | | | |
| **Ly294002 50µM** | 0.5662 | yes | ns | >0.1000 | yes | ns |
| **PD98059 20µM** | 0.0012 | no | ** | | | |
| **U0126 10nM** | 0.8734 | yes | ns | >0.1000 | yes | ns |
| **Erinacine C 5µg/ml 0.5%** | 0.0068 | no | ** | 0.0279 | no | * |
| **K252a 300nM** | 0.7408 | yes | ns | >0.1000 | yes | ns |
| **Bisindolyl-maleimide I 6µM** | 0.737 | yes | ns | | | |
| **Ly294002 50µM** | 0.1776 | yes | ns | >0.1000 | yes | ns |
| **PD98059 20µM** | 0.5601 | yes | ns | >0.1000 | yes | ns |
| **U0126 10nM** | 0.0844 | yes | ns | >0.1000 | yes | ns |

**Table S5:** Test of normal distribution of the values shown in Figure 2C. Normal distribution was tested with Shapiro-Wilk test and Kolmogrov-Smirnov, the p-values were shown (ns not significant $p > 0.05$; * $p < 0.05$; ** $p < 0.01$; *** $p < 0.001$; **** $p < 0.0001$).

| p-values | Shapiro-Wilk test | Normal distribution | Significance | Kolmogorov-Smirnov | Normal distribution | Significance |
|---|---|---|---|---|---|---|
| **Control** | <0.0001 | no | **** | <0.0001 | no | **** |
| **NGF 200ng/ml** | <0.0001 | no | **** | <0.0001 | no | **** |
| **K252a 300nM** | 0.0893 | yes | ns | >0.1000 | yes | ns |
| **Bisindolyl-maleimide I 6µM** | <0.0001 | no | **** | <0.0001 | no | **** |
| **Ly294002 50µM** | 0.0065 | no | ** | >0.1000 | yes | ns |
| **PD98059 20µM** | 0.4207 | yes | ns | >0.1000 | yes | ns |
| **U0126 10nM** | <0.0001 | no | **** | 0.0300 | no | * |
| **Erinacine C 5µg/ml 0.5%** | <0.0001 | no | **** | <0.0001 | no | **** |
| **K252a 300nM** | <0.0001 | no | **** | <0.0001 | no | **** |
| **Bisindolyl-maleimide I 6µM** | 0.0002 | no | *** | 0.0034 | no | ** |
| **Ly294002 50µM** | <0.0001 | no | **** | <0.0001 | no | **** |
| **PD98059 20µM** | 0.0032 | no | ** | 0.0005 | no | *** |
| **U0126 10nM** | <0.0001 | no | **** | <0.0001 | no | **** |

**Table S6:** Test of normal distribution of the values shown in Figure 3. Normal distribution was tested with Shapiro-Wilk test and Kolmogrov-Smirnov, the p-values were shown (ns not significant p > 0.05; * p < 0.05; ** p < 0.01; *** p < 0.001; **** p < 0.0001).

| p-values | Shapiro-Wilk test | Normal distribution | Significance | Kolmogorov-Smirnov | Normal distribution | Significance |
|---|---|---|---|---|---|---|
| **ERE** | <0.0001 | no | **** | <0.0001 | no | **** |
| **FOXO** | <0.0001 | no | **** | <0.0001 | no | **** |
| **NFY** | <0.0001 | no | **** | <0.0001 | no | **** |
| **NBRE** | 0.0004 | no | *** | 0.0055 | no | ** |
| **ETS** | <0.0001 | no | **** | <0.0001 | no | **** |
| **Tbox** | <0.0001 | no | **** | <0.0001 | no | **** |
| **Gli1** | <0.0001 | no | **** | 0.0002 | no | *** |
| **NFkappaB** | <0.0001 | no | **** | <0.0001 | no | **** |
| **STAT3** | 0.0036 | no | ** | >0.1000 | yes | ns |
| **BRE** | <0.0001 | no | **** | <0.0001 | no | **** |
| **HRE** | <0.0001 | no | **** | <0.0001 | no | **** |
| **Elk1** | <0.0001 | no | **** | 0.0408 | no | * |
| **bHLH** | <0.0001 | no | **** | <0.0001 | no | **** |
| **Pea3b** | <0.0001 | no | **** | <0.0001 | no | **** |
| **KLF5** | <0.0001 | no | **** | <0.0001 | no | **** |
| **Dyrk1A** | 0.0011 | no | ** | 0.0045 | no | ** |
| **CREB** | <0.0001 | no | **** | 0.0002 | no | *** |
| **Sim2** | 0.0014 | no | ** | 0.0014 | no | ** |
| **TEAD** | <0.0001 | no | **** | <0.0001 | no | **** |
| **SRE** | 0.2026 | yes | ns | >0.1000 | yes | ns |
| **Lhx2-Sox2** | 0.0003 | no | *** | 0.0022 | no | ** |
| **a1ACT** | 0.0003 | no | *** | 0.0005 | no | *** |
| **Egr1** | <0.0001 | no | **** | <0.0001 | no | **** |
| **bZIP** | <0.0001 | no | **** | <0.0001 | no | **** |
| **HSE** | 0.0002 | no | *** | 0.0085 | no | ** |
| **NRF** | 0.1230 | yes | ns | >0.1000 | yes | ns |
| **Sp1/KLF** | <0.0001 | no | **** | <0.0001 | no | **** |
| **NFAT/AP1** | <0.0001 | no | **** | <0.0001 | no | **** |
| **ARE** | <0.0001 | no | **** | 0.0115 | no | * |
| **E2F** | 0.0011 | no | ** | 0.0102 | no | * |
| **Sox-Pou** | <0.0001 | no | **** | 0.0013 | no | ** |
| **SF1** | <0.0001 | no | **** | <0.0001 | no | **** |
| **RBP7** | <0.0001 | no | **** | <0.0001 | no | **** |
| **TCF/LEF** | <0.0001 | no | **** | 0.0013 | no | ** |
| **SBE** | <0.0001 | no | **** | <0.0001 | no | **** |
| **Runx** | <0.0001 | no | **** | <0.0001 | no | **** |
| **Fexf2** | <0.0001 | no | **** | <0.0001 | no | **** |
| **NFAT** | <0.0001 | no | **** | <0.0001 | no | **** |
| **LexA** | <0.0001 | no | **** | <0.0001 | no | **** |
| **Gal4** | <0.0001 | no | **** | <0.0001 | no | **** |

**Table S7:** Test of normal distribution of the values shown in Figure 4B. Normal distribution was tested with Shapiro-Wilk test and Kolmogrov-Smirnov, the p-values were shown (ns not significant p > 0.05; * p < 0.05; ** p < 0.01; *** p < 0.001; **** p < 0.0001).

| p-values | Shapiro-Wilk test | Normal distribution | Significance | Kolmogorov-Smirnov | Normal distribution | Significance |
|---|---|---|---|---|---|---|
| **control** | 0.0006 | no | *** | 0.0001 | no | *** |
| **EtOH 0.5%** | 0.5234 | yes | ns | >0.1000 | yes | ns |
| **Erinacine C 5µg/ml 0.5%** | 0.0857 | yes | ns | >0.1000 | yes | ns |
| **pCS-ETS1$_{DBD}$-TA4 4xETS-Fluc 1:1** | 0.0650 | yes | ns | 0.0776 | yes | ns |
| **pCS-ETS1$_{DBD}$-TA4 4xETS-Fluc 1:5** | <0.0001 | no | **** | <0.0001 | no | **** |
| **pCS-ETS1$_{DBD}$-TA2 4xETS-Fluc 1:1** | 0.0003 | no | *** | 0.0001 | no | *** |
| **pCS-ETS1$_{DBD}$-TA2 4xETS-Fluc 1:5** | <0.0001 | no | **** | <0.0001 | no | **** |
| **pCS-ETS1$_{DBD}$-KRAB 4xETS-Fluc 1:1** | 0.0005 | no | *** | 0.0007 | no | *** |
| **pCS-ETS1$_{DBD}$-KRAB 4xETS-Fluc 1:5** | 0.0076 | no | ** | 0.0112 | no | * |

**Table S8:** Test of normal distribution of the values shown in Figure 5B. Normal distribution was tested with Shapiro-Wilk test and Kolmogrov-Smirnov, the p-values were shown (ns not significant p > 0.05; * p < 0.05; ** p < 0.01; *** p < 0.001; **** p < 0.0001).

| p-values | Shapiro-Wilk test | Normal distribution | Significance | Kolmogorov-Smirnov | Normal distribution | Significance |
|---|---|---|---|---|---|---|
| **EtOH 0.5%** | 0.2895 | yes | ns | >0.1000 | yes | ns |
| **2x Erinacine C 3µg/ml 0.5%** | 0.926 | yes | ns | >0.1000 | yes | ns |
| **2x Erinacine C 5µg/ml 0.5%** | 0.0014 | no | ** | 0.0389 | no | * |
| **4x Erinacine C 3µg/ml 0.5%** | 0.05 | no | * | >0.1000 | yes | ns |
| **4x Erinacine C 5µg/ml 0.5%** | 0.1327 | yes | ns | >0.1000 | yes | ns |
| **6x Erinacine C 3µg/ml 0.5%** | 0.0005 | no | *** | <0.0001 | no | **** |
| **6x Erinacine C 5µg/ml 0.5%** | 0.1008 | yes | ns | >0.1000 | yes | ns |
| **8x Erinacine C 3µg/ml 0.5%** | 0.0463 | no | * | >0.1000 | yes | ns |
| **8x Erinacine C 5µg/ml 0.5%** | 0.0119 | no | * | 0.0033 | no | ** |

**Table S9:** Test of normal distribution of the values shown in Figure 5C. Normal distribution was tested with Shapiro-Wilk test and Kolmogrov-Smirnov, the p-values were shown (ns not significant p > 0.05; * p < 0.05; ** p < 0.01; *** p < 0.001; **** p < 0.0001).

| p-values | Shapiro-Wilk test | Normal distribution | Significance | Kolmogorov-Smirnov | Normal distribution | Significance |
|---|---|---|---|---|---|---|
| **Control** | 0.0064 | no | ** | 0.0019 | no | ** |
| **EtOH 0.5%** | 0.0024 | no | ** | 0.0221 | no | * |
| **1 µg/ml** | 0.0015 | no | ** | 0.0007 | no | *** |
| **2 µg/ml** | 0.0102 | no | * | 0.0387 | no | * |
| **3 µg/ml** | 0.0415 | no | * | 0.0088 | no | ** |
| **4 µg/ml** | 0.3015 | yes | ns | >0.1000 | yes | ns |
| **5 µg/ml** | 0.0003 | no | *** | <0.0001 | no | **** |
| **10 µg/ml** | 0.0002 | no | *** | <0.0001 | no | **** |
| **15 µg/ml** | 0.484 | yes | ns | >0.1000 | yes | ns |

**Table S10:** Test of normal distribution of the values shown in Figure 5D. Normal distribution was tested with Shapiro-Wilk test and Kolmogrov-Smirnov, the p-values were shown (ns not significant p > 0.05; * p < 0.05; ** p < 0.01; *** p < 0.001; **** p < 0.0001).

| p-values | Shapiro-Wilk test | Normal distribution | Significance | Kolmogorov-Smirnov | Normal distribution | Significance |
|---|---|---|---|---|---|---|
| **Control** | <0.0001 | no | **** | 0.0145 | no | * |
| **EtOH 0.5%** | 0.0023 | no | ** | 0.0002 | no | *** |
| **3h** | 0.0201 | no | * | 0.0173 | no | * |
| **6h** | 0.0465 | no | * | 0.0755 | yes | ns |
| **10h** | 0.0004 | no | *** | 0.0005 | no | *** |
| **16h** | <0.0001 | no | **** | <0.0001 | no | **** |
| **24h** | <0.0001 | no | **** | <0.0001 | no | **** |

**Table S11:** Test of normal distribution of the values shown in Figure 6B. Normal distribution was tested with Shapiro-Wilk test and Kolmogrov-Smirnov, the p-values were shown (ns not significant p > 0.05; * p < 0.05; ** p < 0.01; *** p < 0.001; **** p < 0.0001).

| p-values | Shapiro-Wilk test | Normal distribution | Significance | Kolmogorov-Smirnov | Normal distribution | Significance |
|---|---|---|---|---|---|---|
| **Control *ngf* mRNA** | >0.1000 | yes | ns | >0.1000 | yes | ns |
| **Control *ets1* mRNA** | >0.1000 | yes | ns | >0.1000 | yes | ns |
| **EtOH 0.5% *ngf* mRNA** | 0.2793 | yes | ns | >0.1000 | yes | ns |
| **EtOH 0.5% *ets1* mRNA** | 0.0057 | no | ** | 0.005 | no | ** |
| **3h *ngf* mRNA** | 0.1757 | yes | ns | >0.1000 | yes | ns |
| **3h *ets1* mRNA** | 0.9416 | yes | ns | >0.1000 | yes | ns |
| **6h *ngf* mRNA** | 0.0548 | yes | ns | >0.1000 | yes | ns |
| **6h *ets1* mRNA** | 0.5998 | yes | ns | >0.1000 | yes | ns |
| **10h *ngf* mRNA** | 0.0528 | yes | ns | >0.1000 | yes | ns |
| **10h *ets1* mRNA** | 0.4785 | yes | ns | >0.1000 | yes | ns |
| **16h *ngf* mRNA** | 0.0495 | no | * | >0.1000 | yes | ns |
| **16h *ets1* mRNA** | 0.0688 | yes | ns | >0.1000 | yes | ns |
| **24h *ngf* mRNA** | 0.0370 | no | * | >0.1000 | yes | ns |
| **24h *ets1* mRNA** | 0.7011 | yes | ns | >0.1000 | yes | ns |
| **32h *ngf* mRNA** | 0.0288 | no | * | >0.1000 | yes | ns |
| **32h *ets1* mRNA** | 0.7249 | yes | ns | >0.1000 | yes | ns |
| **48h *ngf* mRNA** | 0.1740 | yes | ns | >0.1000 | yes | ns |
| **48h *ets1* mRNA** | 0.7312 | yes | ns | >0.1000 | yes | ns |

**Table S12:** Test of normal distribution of the values shown in Figure 6C. Normal distribution was tested with Shapiro-Wilk test and Kolmogrov-Smirnov, the p-values were shown (ns not significant p > 0.05; * p < 0.05; ** p < 0.01; *** p < 0.001; **** p < 0.0001).

| p-values | Shapiro-Wilk test | Normal distribution | Significance | Kolmogorov-Smirnov | Normal distribution | Significance |
|---|---|---|---|---|---|---|
| **Control 24h** | >0.1000 | yes | ns | >0.1000 | yes | ns |
| **pCS-ETS$_{DBD}$-TA4 24h** | 0.9977 | yes | ns | >0.1000 | yes | ns |
| **pCS-ETS$_{DBD}$-TA2 24h** | 0.9901 | yes | ns | >0.1000 | yes | ns |
| **Control 48h** | >0.1000 | yes | ns | >0.1000 | yes | ns |
| **pCS-ETS$_{DBD}$-TA4 48h** | 0.9941 | yes | ns | >0.1000 | yes | ns |
| **pCS-ETS$_{DBD}$-TA2 48h** | 0.9968 | yes | ns | >0.1000 | yes | ns |

**Table S13:** Test of normal distribution of the values shown in Figure 7A. Normal distribution was tested with Shapiro-Wilk test and Kolmogrov-Smirnov, the p-values were shown (ns not significant p > 0.05; * p < 0.05; ** p < 0.01; *** p < 0.001; **** p < 0.0001).

| p-values | Shapiro-Wilk test | Normal distribution | Significance | Kolmogorov-Smirnov | Normal distribution | Significance |
|---|---|---|---|---|---|---|
| **EtOH 0.5% *ngf* mRNA** | <0.0001 | no | **** | <0.0001 | no | **** |
| **EtOH 0.5% *bdnf* mRNA** | <0.0001 | no | **** | <0.0001 | no | **** |
| **Erinacine C *ngf* mRNA** | 0.0166 | no | * | 0.084 | yes | ns |
| **Erinacine C *bdnf* mRNA** | 0.0465 | no | * | >0.1000 | yes | ns |
| **Erinacine C + PD98059 *ngf* mRNA** | 0.4177 | yes | ns | >0.1000 | yes | ns |
| **Erinacine C + PD98059 *bdnf* mRNA** | 0.1053 | yes | ns | >0.1000 | yes | ns |
| **Erinacine C + U0126 *ngf* mRNA** | 0.1601 | yes | ns | >0.1000 | yes | ns |
| **Erinacine C + U0126 *bdnf* mRNA** | 0.9806 | yes | ns | >0.1000 | yes | ns |
| **Erinacine C + PD98059 + U0126 *ngf* mRNA** | 0.1604 | yes | ns | >0.1000 | yes | ns |
| **Erinacine C + PD98059 + U0126 *bdnf* mRNA** | 0.8963 | yes | ns | >0.1000 | yes | ns |

**Table S14:** Test of normal distribution of the values shown in Figure 7B. Normal distribution was tested with Shapiro-Wilk test and Kolmogrov-Smirnov, the p-values were shown (ns not significant p > 0.05; * p < 0.05; ** p < 0.01; *** p < 0.001; **** p < 0.0001).

| p-values | Shapiro-Wilk test | Normal distribution | Significance | Kolmogorov-Smirnov | Normal distribution | Significance |
|---|---|---|---|---|---|---|
| **EtOH *ngf* mRNA** | >0.1000 | yes | ns | >0.1000 | yes | ns |
| **EtOH *bdnf* mRNA** | >0.1000 | yes | ns | >0.1000 | yes | ns |
| **Vehicle *ngf* mRNA** | 0.8262 | yes | ns | >0.1000 | yes | ns |
| **Vehicle *bdnf* mRNA** | 0.6041 | yes | ns | >0.1000 | yes | ns |
| **pCS-ETS$_{DBD}$-KRAB *ngf* mRNA** | 0.9739 | yes | ns | >0.1000 | yes | ns |
| **pCS-ETS$_{DBD}$-KRAB *bdnf* mRNA** | 0.2189 | yes | ns | >0.1000 | yes | ns |

**Table S15:** Test of statistical significance of the values shown in Figure 1E. Statistical significance was tested with either One-way ANOVA with Bonferroni (when normally distributed) or with One-way ANOVA Kruskal-Wallis (when not normally distributed), the p-values were shown (ns not significant p > 0.05; * p < 0.05; ** p < 0.01; *** p < 0.001; **** p < 0.0001).

| p-values | Bonferroni | Significance | Kruskal-Wallis | Significance |
|---|---|---|---|---|
| **control** | | | | |
| **untransfected + NGF 200ng/ml** | | | <0.0001 To control | **** To control |
| **Fyntag mCitrine + NGF 200ng/ml** | | | <0.0001 To control | **** To control |
| **dnTrkA + NGF 200ng/ml** | | | 0.0013 To control | ** To control |
| | | | <0.0001 To untransfected + NGF | **** To untransfected + NGF |
| | | | <0.0001 To Fyntag mCirtrine + NGF | **** To Fyntag mCirtrine + NGF |
| **EtOH 0.5%** | | | 0.7772 To control | ns To control |
| **Untransfected + Erinacine C 5µg/ml 0.5%** | | | <0.0001 To control | **** To control |
| **Fyntag mCitrine + Erinacine C 5µg/ml 0.5%** | | | <0.0001 To EtOH | **** To EtOH |
| **dnTrkA + Erinacine C 5µg/ml 0.5%** | | | 0.0226 To EtOH | * To EtOH |
| | | | 0.0001 To untransfected + Erinacine C | *** To untransfected + Erinacine C |
| | | | <0.0001 To Fyntag mCritrine + Erinacine | **** To Fyntag mCitrine + Erinacine |

**Table S16:** Test of statistical significance of the values shown in Figure 1F. Statistical significance was tested with either One-way ANOVA with Bonferroni (when normally distributed) or with One-way ANOVA Kruskal-Wallis (when not normally distributed), the p-values were shown (ns not significant p > 0.05; * p < 0.05; ** p < 0.01; *** p < 0.001; **** p < 0.0001).

| p-values | Bonferroni | Significance | Kruskal-Wallis | Significance |
|---|---|---|---|---|
| **control** | | | | |
| **untransfected + NGF 200ng/ml** | | | <0.0001<br>To control | ****<br>To control |
| **Fyntag mCitrine + NGF 200ng/ml** | | | <0.0001<br>To control | ****<br>To control |
| **dnTrkA + NGF 200ng/ml** | | | <0.0001<br>To control | ****<br>To control |
| | | | <0.0001<br>To untransfected + NGF | ****<br>To untransfected + NGF |
| | | | <0.0001<br>To Fyntag mCirtrine + NGF | ****<br>To Fyntag mCirtrine + NGF |
| **EtOH 0.5%** | | | 0.8337<br>To control | ns<br>To control |
| **Untransfected + Erinacine C 5µg/ml 0.5%** | | | <0.0001<br>To control | ****<br>To control |
| **Fyntag mCitrine + Erinacine C 5µg/ml 0.5%** | | | <0.0001<br>To EtOH | ****<br>To EtOH |
| **dnTrkA + Erinacine C 5µg/ml 0.5%** | | | <0.0001<br>To EtOH | ****<br>To EtOH |
| | | | <0.0001<br>To untransfected + Erinacine C | ****<br>To untransfected + Erinacine C |
| | | | <0.0001<br>To Fyntag mCritrine + Erinacine | ****<br>To Fyntag mCitrine + Erinacine |

**Table S17:** Test of statistical significance of the values shown in Figure 2B. Statistical significance was tested with either One-way ANOVA with Bonferroni (when normally distributed) or with One-way ANOVA Kruskal-Wallis (when not normally distributed), the p-values were shown (ns not significant p > 0.05; * p < 0.05; ** p < 0.01; *** p < 0.001; **** p < 0.0001).

| p-values | Bonferroni | Significance | Kruskal-Wallis | Significance |
|---|---|---|---|---|
| **Control** | | | | |
| **NGF 200ng/ml** | | | <0.0001<br>To control | **** |
| **K252a 300nM** | 0.0322<br>To NGF | * | | |
| **Bisindolylmaleimide I 6µM** | 0.0008<br>To NGF | *** | | |
| **Ly294002 50µM** | 0.0455<br>To NGF | * | | |
| **PD98059 20µM** | | | 0.0075<br>To NGF | ** |
| **U0126 10nM** | 0.0045<br>To NGF | ** | | |
| **Erinacine C 5µg/ml 0.5%** | | | 0.0075<br>To control | ** |
| **K252a 300nM** | | | 0.0333<br>To Erinacine | * |
| **Bisindolylmaleimide I 6µM** | | | 0.0487<br>To Erinacine | * |
| **Ly294002 50µM** | | | 0.0301<br>To Erinacine | * |
| **PD98059 20µM** | | | 0.0322<br>To Erinacine | * |
| **U0126 10nM** | | | 0.0318<br>To Erinacine | * |

**Table S18:** Test of statistical significance of the values shown in Figure 2C. Statistical significance was tested with either One-way ANOVA with Bonferroni (when normally distributed) or with One-way ANOVA Kruskal-Wallis (when not normally distributed), the p-values were shown (ns not significant p > 0.05; * p < 0.05; ** p < 0.01; *** p < 0.001; **** p < 0.0001).

| p-values | Bonferroni | Significance | Kruskal-Wallis | Significance |
|---|---|---|---|---|
| **Control** | | | | |
| **NGF 200ng/ml** | | | <0.0001<br>to control | **** |
| **K252a 300nM** | | | 0.0014<br>To NGF | ** |
| **Bisindolylmaleimide I 6µM** | | | <0.0001<br>To NGF | **** |
| **Ly294002 50µM** | | | 0.0022<br>To NGF | ** |
| **PD98059 20µM** | | | 0.0002<br>To NGF | *** |
| **U0126 10nM** | | | 0.0019<br>To NGF | ** |
| **Erinacine C 5µg/ml 0.5%** | | | 0.00088<br>To control | *** |
| **K252a 300nM** | | | 0.0087<br>To Erinacine | ** |
| **Bisindolylmaleimide I 6µM** | | | 0.0221<br>To Erinacine | * |
| **Ly294002 50µM** | | | 0.0012<br>To Erinacine | ** |
| **PD98059 20µM** | | | 0.0001<br>To Erinacine | *** |
| **U0126 10nM** | | | 0.0015<br>To Erinacine | ** |

**Table S19:** Test of statistical significance of the values shown in Figure 3. Statistical significance was tested with either One-way ANOVA with Bonferroni (when normally distributed) or with One-way ANOVA Kruskal-Wallis (when not normally distributed), the p-values were shown (ns not significant $p > 0.05$; * $p < 0.05$; ** $p < 0.01$; *** $p < 0.001$; **** $p < 0.0001$).

| p-values | Bonferroni | Significance | Kruskal-Wallis | Significance |
|---|---|---|---|---|
| **ERE** | | | 0.0357 | * |
| **FOXO** | | | 0.4245 | ns |
| **NFY** | | | 0.3863 | ns |
| **NBRE** | | | 0.1715 | ns |
| **ETS** | | | 0.0412 | * |
| **Tbox** | | | 0.3525 | ns |
| **Gli1** | | | 0.4779 | ns |
| **NFkappaB** | | | 0.7054 | ns |
| **STAT3** | | | 0.0882 | ns |
| **BRE** | | | 0.9690 | ns |
| **HRE** | | | 0.9152 | ns |
| **Elk1** | | | 0.0510 | ns |
| **bHLH** | | | 0.1437 | ns |
| **Pea3b** | | | 0.1929 | ns |
| **KLF5** | | | 0.8990 | ns |
| **Dyrk1A** | | | 0.9894 | ns |
| **CREB** | | | >0.9999 | ns |
| **Sim2** | | | 0.7437 | ns |
| **TEAD** | | | 0.7170 | ns |
| **SRE** | | | 0.6558 | ns |
| **Lhx2-Sox2** | | | 0.7131 | ns |
| **a1ACT** | | | >0.9999 | ns |
| **Egr1** | | | 0.2062 | ns |
| **bZIP** | | | 0.3613 | ns |
| **HSE** | | | 0.2797 | ns |
| **NRF** | | | 0.7691 | ns |
| **Sp1/KLF** | | | 0.9010 | ns |
| **NFAT/AP1** | | | 0.8587 | ns |
| **ARE** | | | 0.7996 | ns |
| **E2F** | | | 0.6362 | ns |
| **Sox-Pou** | | | 0.0885 | ns |
| **SF1** | | | 0.2647 | ns |
| **RBP7** | | | 0.2647 | ns |
| **TCF/LEF** | | | 0.0885 | ns |
| **SBE** | | | >0.9999 | ns |
| **Runx** | | | >0.9999 | ns |
| **Fexf2** | | | >0.9999 | ns |
| **NFAT** | | | >0.9999 | ns |
| **LexA** | | | >0.9999 | ns |
| **Gal4** | | | >0.9999 | ns |

**Table S20:** Test of statistical significance of the values shown in Figure 4B. Statistical significance was tested with either One-way ANOVA with Bonferroni (when normally distributed) or with One-way ANOVA Kruskal-Wallis (when not normally distributed), the p-values were shown (ns not significant p > 0.05; * p < 0.05; ** p < 0.01; *** p < 0.001; **** p < 0.0001).

| p-values | Bonferroni | Significance | Kruskal-Wallis | Significance |
|---|---|---|---|---|
| **control** | | | | |
| **EtOH 0.5%** | | | 0.8699 | ns |
| **Erinacine C 5µg/ml 0.5%** | | | 0.046 | * |
| **pCS-ETS1$_{DBD}$-TA4 4xETS-Fluc 1:1** | | | 0.181 | ns |
| **pCS-ETS1$_{DBD}$-TA4 4xETS-Fluc 1:5** | | | 0.02 | * |
| **pCS-ETS1$_{DBD}$-TA2 4xETS-Fluc 1:1** | | | 0.0504 | ns |
| **pCS-ETS1$_{DBD}$-TA2 4xETS-Fluc 1:5** | | | 0.0341 | * |
| **pCS-ETS1$_{DBD}$-KRAB 4xETS-Fluc 1:1** | | | 0.0006 | *** |
| **pCS-ETS1$_{DBD}$-KRAB 4xETS-Fluc 1:5** | | | 0.0015 | ** |

**Table S21:** Test of statistical significance of the values shown in Figure 5B. Statistical significance was tested with either One-way ANOVA with Bonferroni (when normally distributed) or with One-way ANOVA Kruskal-Wallis (when not normally distributed), the p-values were shown (ns not significant p > 0.05; * p < 0.05; ** p < 0.01; *** p < 0.001; **** p < 0.0001).

| p-values | Bonferroni | Significance | Kruskal-Wallis | Significance |
|---|---|---|---|---|
| **EtOH 0.5%** | | | | |
| **2x Erinacine C 3µg/ml 0.5%** | | | 0.525 | ns |
| **2x Erinacine C 5µg/ml 0.5%** | | | 0.1672 | ns |
| **4x Erinacine C 3µg/ml 0.5%** | | | 0.023 | * |
| **4x Erinacine C 5µg/ml 0.5%** | | | 0.0376 | * |
| **6x Erinacine C 3µg/ml 0.5%** | | | 0.0003 | *** |
| **6x Erinacine C 5µg/ml 0.5%** | | | 0.0008 | *** |
| **8x Erinacine C 3µg/ml 0.5%** | | | <0.0001 | **** |
| **8x Erinacine C 5µg/ml 0.5%** | | | 0.0005 | *** |

**Table S22:** Test of statistical significance of the values shown in Figure 5C. Statistical significance was tested with either One-way ANOVA with Bonferroni (when normally distributed) or with One-way ANOVA Kruskal-Wallis (when not normally distributed), the p-values were shown (ns not significant p > 0.05; * p < 0.05; ** p < 0.01; *** p < 0.001; **** p < 0.0001).

| p-values | Bonferroni | Significance | Kruskal-Wallis | Significance |
|---|---|---|---|---|
| **Control** | | | | |
| **EtOH 0.5%** | | | | |
| **1 µg/ml** | | | 0.6764 | ns |
| **2 µg/ml** | | | 0.5156 | ns |
| **3 µg/ml** | | | 0.1066 | ns |
| **4 µg/ml** | | | 0.0314 | * |
| **5 µg/ml** | | | 0.3462 | ns |
| **10 µg/ml** | | | 0.7835 | ns |
| **15 µg/ml** | | | 0.0054 | ** |

**Table S23:** Test of statistical significance of the values shown in Figure 5D. Statistical significance was tested with either One-way ANOVA with Bonferroni (when normally distributed) or with One-way ANOVA Kruskal-Wallis (when not normally distributed), the p-values were shown (ns not significant p > 0.05; * p < 0.05; ** p < 0.01; *** p < 0.001; **** p < 0.0001).

| p-values | Bonferroni | Significance | Kruskal-Wallis | Significance |
|---|---|---|---|---|
| **Control** | | | | |
| **EtOH 0.5%** | | | 0.9454 | ns |
| **3h** | | | 0.4686 | ns |
| **6h** | | | 0.3134 | ns |
| **10h** | | | 0.8791 | ns |
| **16h** | | | 0.7304 | ns |
| **24h** | | | 0.0018 | ** |

**Table S24:** Test of statistical significance of the values shown in Figure 6B. Statistical significance was tested with either One-way ANOVA with Bonferroni (when normally distributed) or with One-way ANOVA Kruskal-Wallis (when not normally distributed), the p-values were shown (ns not significant p > 0.05; * p < 0.05; ** p < 0.01; *** p < 0.001; **** p < 0.0001).

| p-values | Bonferroni | Significance | Kruskal-Wallis | Significance |
|---|---|---|---|---|
| **Control *ngf* mRNA** | | | | |
| **Control *ets1* mRNA** | | | | |
| **EtOH 0.5% *ngf* mRNA** | >0.9999 To control | ns | | |
| **EtOH 0.5% *ets1* mRNA** | | | 0.6378 To control | ns |
| **3h *ngf* mRNA** | >0.9999 To control | ns | | |
| | >0.9999 To EtOH | ns | | |
| **3h *ets1* mRNA** | >0.9999 To control | ns | 0.8564 To EtOH | ns |
| **6h *ngf* mRNA** | >0.9999 To control | ns | | |
| | >0.9999 To EtOH | ns | | |
| **6h *ets1* mRNA** | <0.0001 To control | **** | 0.0113 To EtOH | * |
| **10h *ngf* mRNA** | >0.9999 To control | ns | | |
| | >0.9999 To EtOH | ns | | |
| **10h *ets1* mRNA** | >0.9999 To control | ns | 0.0335 To EtOH | * |
| **16h *ngf* mRNA** | | | 0.0982 To control | ns |
| | | | 0.0982 To EtOH | ns |
| **16h *ets1* mRNA** | >0.9999 To control | ns | 0.3902 To EtOH | ns |
| **24h *ngf* mRNA** | | | 0.0229 To control | * |
| | | | 0.0229 To EtOH | * |
| **24h *ets1* mRNA** | >0.9999 To control | ns | 0.3657 To EtOH | ns |
| **32h *ngf* mRNA** | | | 0.0036 To control | ** |
| | | | 0.0036 To EtOH | ** |
| **32h *ets1* mRNA** | <0.0001 To control | **** | 0.0335 To EtOH | * |
| **48h *ngf* mRNA** | 0.0060 To control | ** | | |
| | 0.0068 To EtOH | ** | | |
| **48h *ets1* mRNA** | <0.0001 To control | **** | 0.0231 To EtOH | * |

**Table S25:** Test of statistical significance of the values shown in Figure 6C. Statistical significance was tested with either One-way ANOVA with Bonferroni (when normally distributed) or with One-way ANOVA Kruskal-Wallis (when not normally distributed), the p-values were shown (ns not significant p > 0.05; * p < 0.05; ** p < 0.01; *** p < 0.001; **** p < 0.0001).

| p-values | Bonferroni | Significance | Kruskal-Wallis | Significance |
|---|---|---|---|---|
| **Control 24h** | | | | |
| **pCS-ETS$_{DBD}$-TA4 24h** | >0.9999 | ns | | |
| **pCS-ETS$_{DBD}$-TA2 24h** | >0.9999 | ns | | |
| **Control 48h** | | | | |
| **pCS-ETS$_{DBD}$-TA4 48h** | >0.9999 | ns | | |
| **pCS-ETS$_{DBD}$-TA2 48h** | >0.9999 | ns | | |

**Table S26:** Test of statistical significance of the values shown in Figure 7A. Statistical significance was tested with either One-way ANOVA with Bonferroni (when normally distributed) or with One-way ANOVA Kruskal-Wallis (when not normally distributed), the p-values were shown (ns not significant p > 0.05; * p < 0.05; ** p < 0.01; *** p < 0.001; **** p < 0.0001).

| p-values | Bonferroni | Significance | Kruskal-Wallis | Significance |
|---|---|---|---|---|
| **EtOH 0.5%<br>*ngf* mRNA** | | | | |
| **EtOH 0.5%<br>*bdnf* mRNA** | | | | |
| **Erinacine C<br>*ngf* mRNA** | | | <0.0001<br>To EtOH | **** |
| **Erinacine C<br>*bdnf* mRNA** | | | 0.0383<br>To EtOH | * |
| **Erinacine C +<br>PD98059<br>*ngf* mRNA** | 0.8219<br>To Erinacine | ns | 0.0027<br>To EtOH | ** |
| **Erinacine C +<br>PD98059<br>*bdnf* mRNA** | 0.8074<br>To Erinacine | ns | 0.0188<br>To EtOH | * |
| **Erinacine C + U0126<br>*ngf* mRNA** | 0.6735<br>To Erinacine | ns | 0.0080<br>To EtOH | ** |
| **Erinacine C + U0126<br>*bdnf* mRNA** | 0.4807<br>To Erinacine | ns | 0.0099<br>To EtOH | ** |
| **Erinacine C +<br>PD98059 + U0126<br>*ngf* mRNA** | 0.8149<br>To Erinacine | ns | 0.0045<br>To EtOH | ** |
| **Erinacine C +<br>PD98059 + U0126<br>*bdnf* mRNA** | 0.2885<br>To Erinacine | ns | 0.5307<br>To EtOH | ns |

**Table S27:** Test of statistical significance of the values shown in Figure 7B. Statistical significance was tested with either One-way ANOVA with Bonferroni (when normally distributed) or with One-way ANOVA Kruskal-Wallis (when not normally distributed), the p-values were shown (ns not significant p > 0.05; * p < 0.05; ** p < 0.01; *** p < 0.001; **** p < 0.0001).

| p-values | Bonferroni | Significance | Kruskal-Wallis | Significance |
|---|---|---|---|---|
| **EtOH *ngf* mRNA** | | | | |
| **EtOH *bdnf* mRNA** | | | | |
| **Vehicle *ngf* mRNA** | 0.0073<br>To EtOH | ** | | |
| **Vehicle *bdnf* mRNA** | 0.049<br>To EtOH | * | | |
| **pCS-ETS$_{DBD}$-KRAB *ngf* mRNA** | <0.0001<br>To EtOH | **** | | |
| | >0.9999<br>To Vehicle | ns | | |
| **pCS-ETS$_{DBD}$-KRAB *bdnf* mRNA** | 0.041<br>To EtOH | * | | |
| | >0.9999<br>To Vehicle | ns | | |

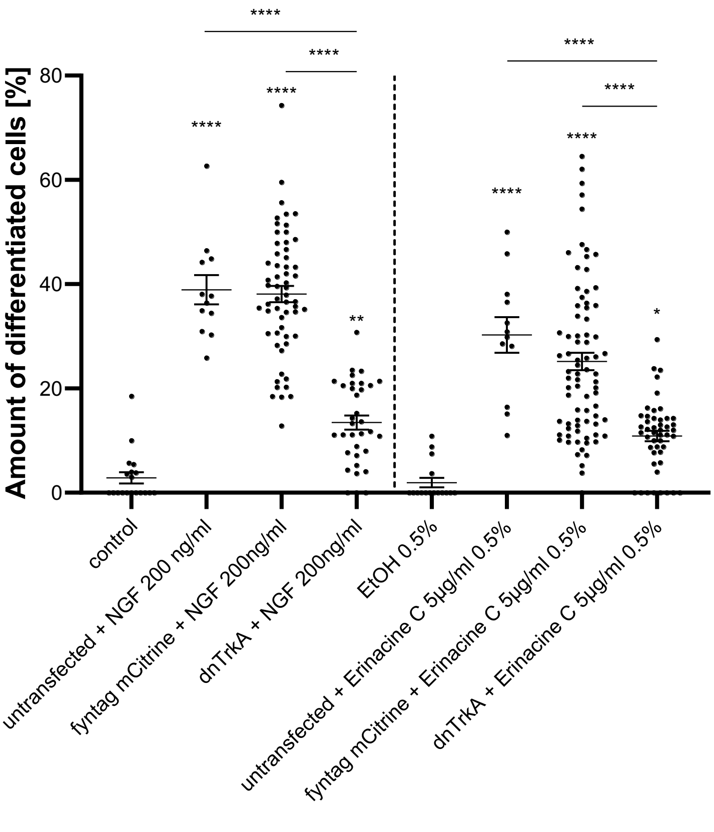


**Figure S1:** Scatter plot to Figure 1E. Points show individual values of the experiment.

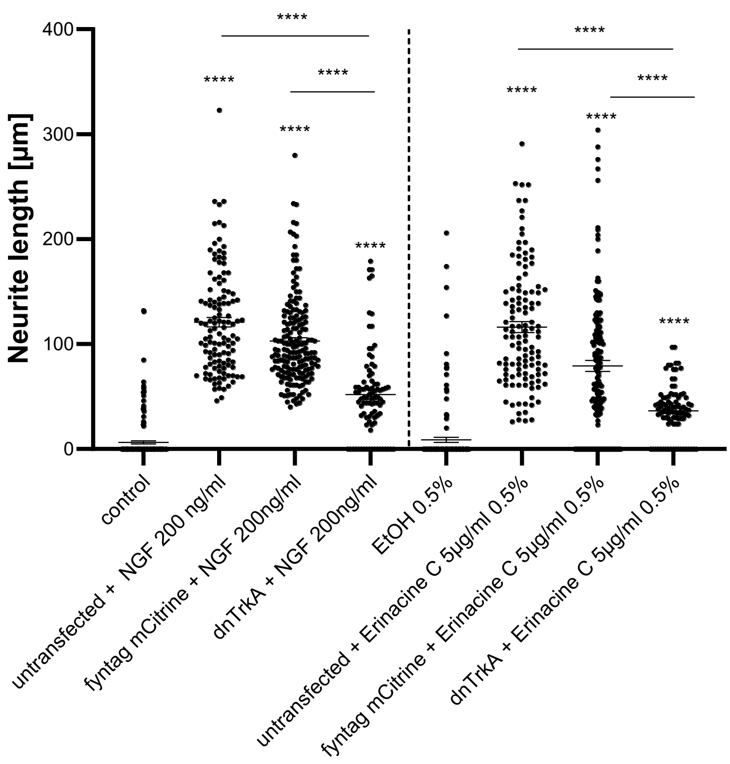


**Figure S2:** Scatter plot to Figure 1F. Points show individual values of the experiment.

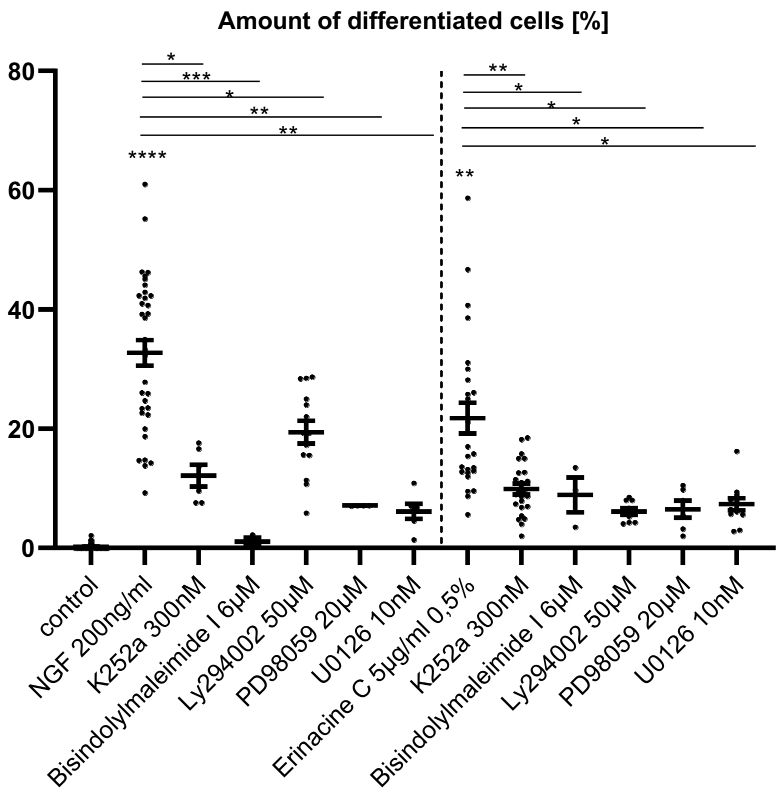


**Figure S3:** Scatter plot to Figure 2B. Points show individual values of the experiment.

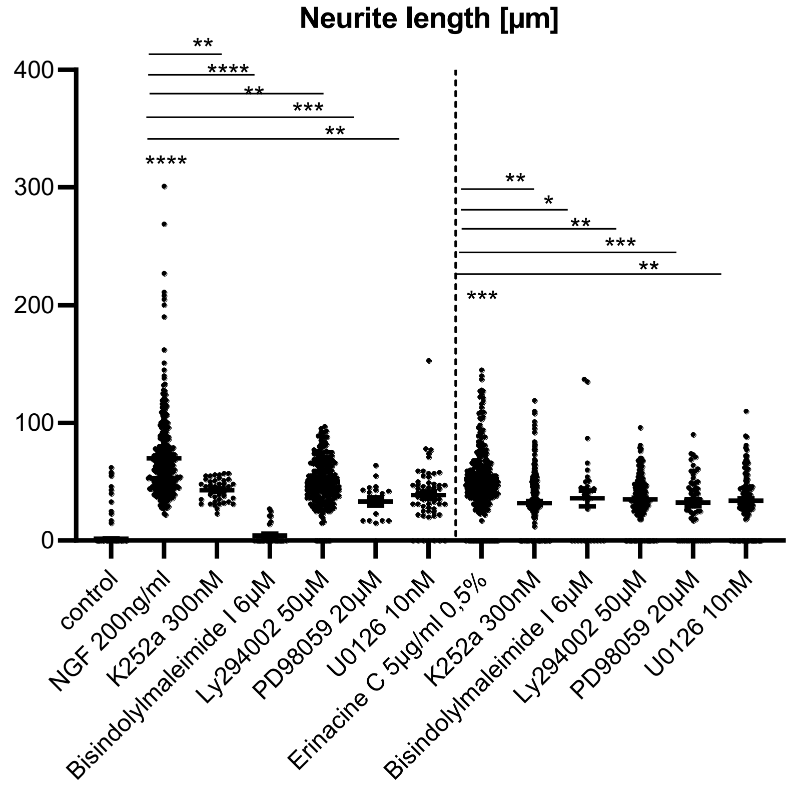


**Figure S4:** Scatter plot to Figure 2C. Points show individual values of the experiment.

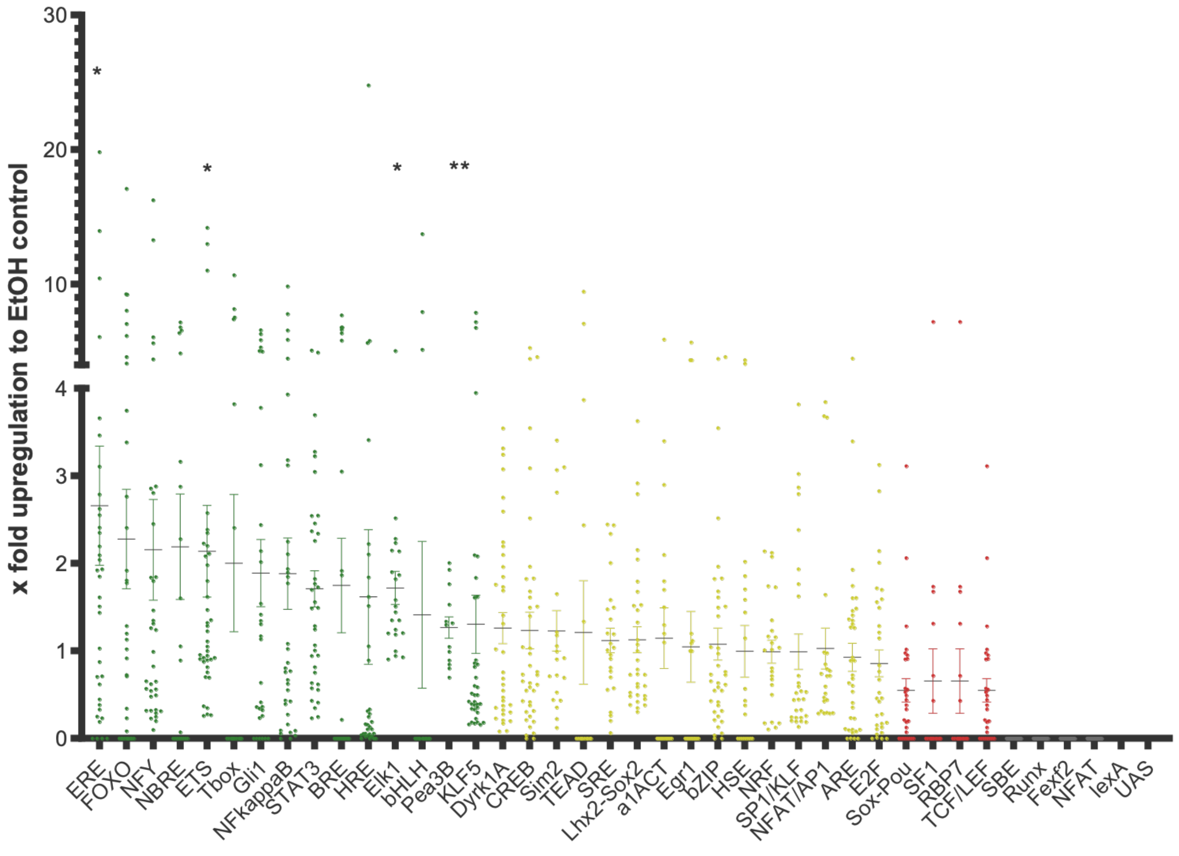


**Figure S5:** Scatter plot to Figure 3. Points show individual values of the experiment.

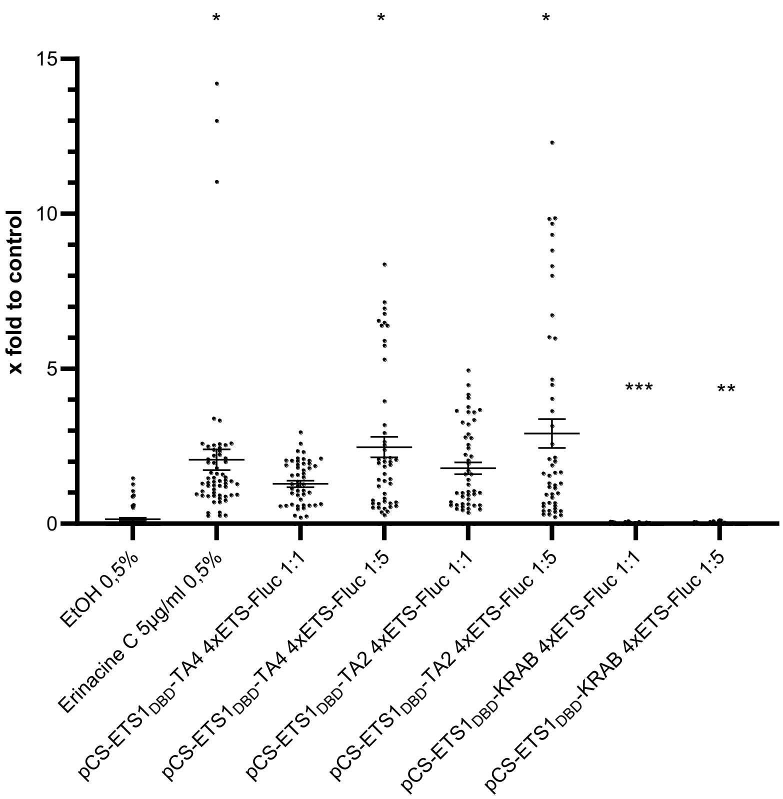


**Figure S6:** Scatter plot to Figure 4B. Points show individual values of the experiment.

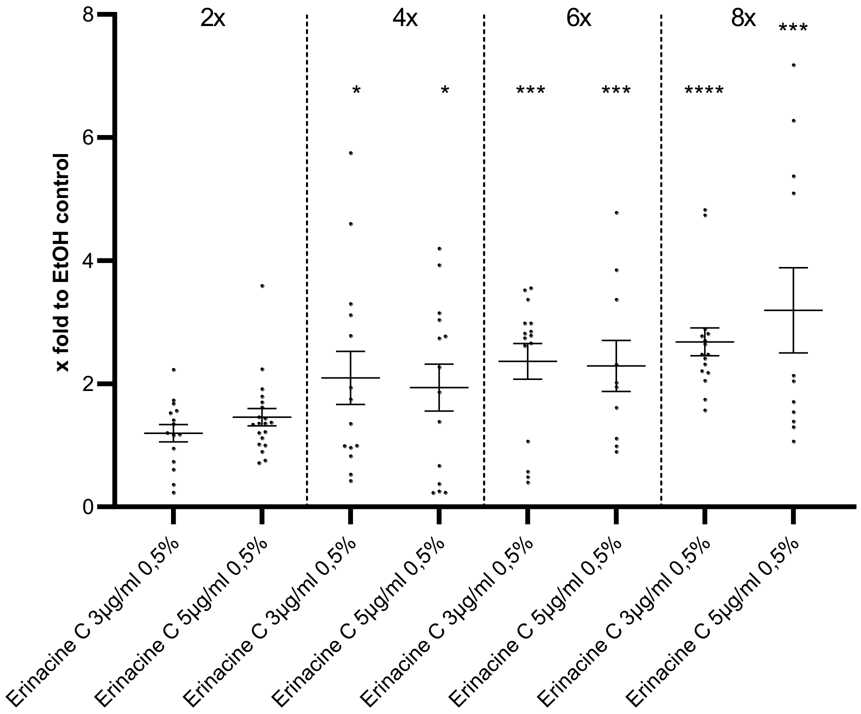


**Figure S7:** Scatter plot to Figure 5B. Points show individual values of the experiment.

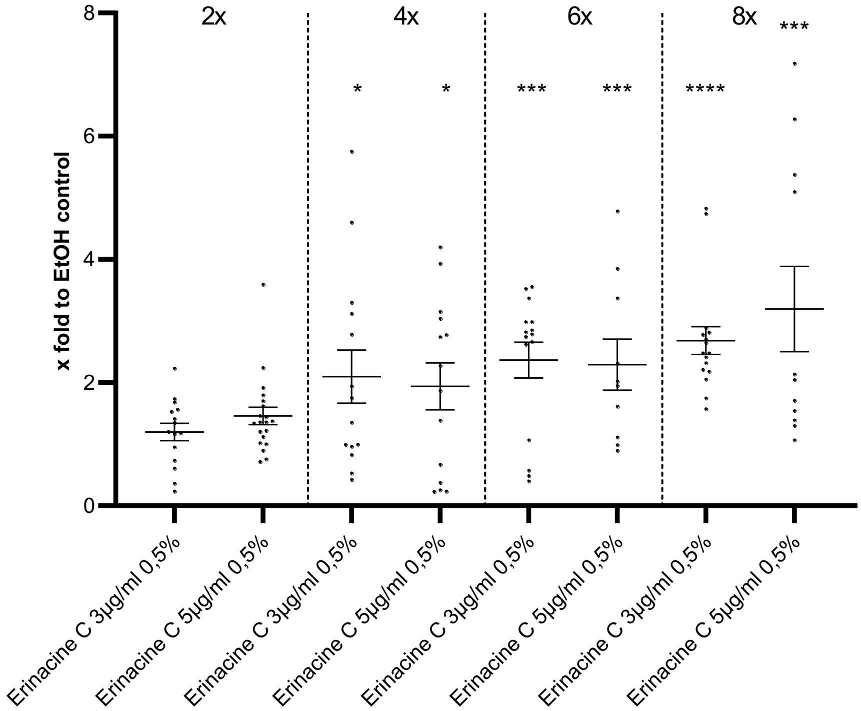


**Figure S8:** Scatter plot to Figure 5C. Points show individual values of the experiment.

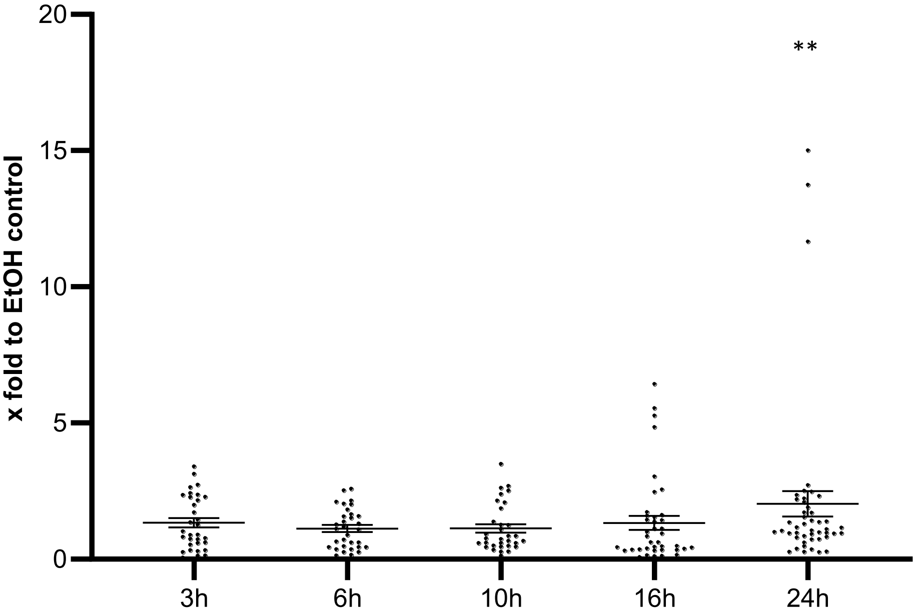


**Figure S9:** Scatter plot to Figure 5D. Points show individual values of the experiment.

**Table S28:** List of Firefly luciferase constructs containing 4 transcription factor DNA binding sites

| DNA-binding site (below: sense oligo used for cloning) | Signaling pathway | Construct number |
|---|---|---|
| **α1ACT**, [1] | α1ACT signaling (C-terminus of CACNA1a) | #5460 |
| GGTACCTCTAGAATTATAAGATGTCTGATATTATAAGATGCTCGAAATTATAAGATGTCTGATATTATAAGATGCTCGAGCCTGCAGG | | |
| **ARE**, [2] | anti-oxidant electrophile responsive element | #5290 |
| GGTACCTCTAGAGGAAATGACATTGCTAATGGTGACAAAGCAACTTTTCTGATGGAAATGACATTGCTAATGGTGACAAAGCAACTTTCTCGAAGGAAATGACATTGCTAATGGTGACAAAGCAACTTTCTCGAGCCTGCAGG (cloning resulted in 4x ARE bdg. sites) | | |
| **bHLH**, E-box, [3] | bHLH transcription factors | #5297 |
| GGTACCTCTAGAAAACAGCTGTTCTGATAAACAGCTGTCTCGAAAAACAGCTGTTCTGATAAACAGCTGTCTCGAGCCTGCAGG | | |
| **BRE**, [4] | BMP signaling | #5347 |
| GGTACCTCTAGACTGGCGCCTCTGATCTGGAGCCCTCGAACTGGCGCCTCTGATCTGGAGCCCTCGAGCCTGCAGG | | |
| **bZIP**, [3] | basic leucine zipper transcription factors | #5298 |
| GGTACCTCTAGAGATGAGTCATCCTCTGATGATGAGTCATCCCTCGAAGATGAGTCATCCTCTGATGATGAGTCATCCCTCGAGCCTGCAGG | | |
| **CREB**, [5,6] | c-AMP signaling | #5273 |
| GGTACCTCTAGACTGACGTCATCTGATCTGACGTCACTCGAACTGACGTCATCTGATCTGACGTCACTCGAGCCTGCAGG | | |
| **Dyrk1A**, (Di Vona et al., 2015) | Dyrk1A kinase signaling | #5270 |
| GGTACCTCTAGATCTCGCGAGATCTGATTCTCGCGAGACTCGAATCTCGCGAGATCTGATTCTCGCGAGACTCGAGCCTGCAGG | | |
| **E2F**, [3] | E2F transcription factors | #5296 |
| GGTACCTCTAGACTGGCGGGAATCTGATCTGGCGGGAACTCGAACTGGCGGGAATCTGATCTGGCGGGAACTCGAGCCTGCAGG | | |
| **Egr1** (NGFI-A), [8] | Egr-1 transcription factor | #5304 |
| GGTACCTCTAGACTCCCCCACTCTGATCGCCCCCGCCTCGAACTCCCCCACTCTGATCGCCCCCGCCTCGAGCCTGCAGG | | |
| **Elk1**, [9–11] | MAPK signaling | #5291 |
| GGTACCTCTAGAGACCGGAAGTTCTGATAACCGGAAGTCTCGAAGACCGGAAGTTCTGATAACCGGAAGTCTCGAGCCTGCAGG | | |
| **ERE**, [12] | estrogen signaling | #5306 |
| GGTACCTCTAGAGGTCACAGTGACCTCTGATGGTCACAGTGACCCTCGAAGGTCACAGTGACCTCTGATGGTCACAGTGACCCTCGAGCCTGCAGG | | |
| **ETS**, [3] | MAPK signaling | #5301 |
| GGTACCTCTAGACACTTCCGGTTCTGATCACTTCCGGTCTCGAACACTTCCGGTTCTGATCACTTCCGGTCTCGAGCCTGCAGG | | |
| **Fezf2**, [13] | Fez signaling | #5308 |
| GGTACCTCTAGACAGCAACCTCTGATCAGCAACCCTCGAACAGCAACCTCTGATCAGCAACCCTCGAGCCTGCAGG | | |

| | | |
|---|---|---|
| **FOXO**, [3] | Forkhead box protein signaling | #5346 |
| GGTACCTCTAGATCCTGTTTACCATCTGATTCCTGTTTACCACTCGAATCCTGTTTACCATCTGATTCCTGTTTACCACTCGAGCCTGCAGG | | |
| **Gal4**, [14,15] | yeast Gal4 operon regulation | #5288 |
| GGTACCTCTAGACGGAGTACTGTCCTCCGTCTGATCGGAGTACTGTCCTCCGCTCGAACGGAGTACTGTCCTCCGTCTGATCGGAGTACTGTCCTCCGCTCGAGCCTGCAGG | | |
| **Gli-1**, [16,17] | Hedgehog signaling | #5266 |
| GGTACCTCTAGAGACCACCCATCTGATGACCACCCACTCGAAGACCACCCATCTGATGACCACCCACTCGAGCCTGCAGG | | |
| **HRE**, [18,19] | hypoxia induced signaling | #5277 |
| GGTACCTCTAGATGTGTACGTGCTGTCTGATTGTGTACGTGCTGCTCGAATGTGTACGTGCTGTCTGATTGTGTACGTGCTGCTCGAGCCTGCAGG | | |
| **HSE**, [20] | heat-shock induced signaling | #5281 |
| GGTACCTCTAGAAGAACGTTCTAGAACTCTGATAGAACGTTCTAGAACCTCGAAAGAACGTTCTAGAACTCTGATAGAACGTTCTAGAACCTCGAGCCTGCAGG | | |
| **KLF5**, [3] | Krüppel-like transcription factors | #5300 |
| GGTACCTCTAGAAGGGTGTGGCTCTGATAGGGTGTGGCCTCGAAAGGGTGTGGCTCTGATAGGGTGTGGCCTCGAGCCTGCAGG | | |
| **LexA**, [21] | bacterial lex operon regulation | #5342 |
| GGTACCTCTAGACTGTACATCCATACAGTCTGATCTGTACATCCATACAGCTCGAACTGTACATCCATACAGTCTGATCTGTACATCCATACAGCTCGAGCCTGCAGG | | |
| **Lhx2-Sox2**, [22,23] | Lhx-Sox transcription factors | #5275 |
| GGTACCTCTAGACTAATTAAAGAACAAAGTCTGATCTAATTAAAGAACAAAGCTCGAACTAATTAAAGAACAAAGTCTGATCTAATTAAAGAACAAAGCTCGAGCCTGCAGG | | |
| **NBRE** (NGFI-B), [24–26] | steroid and BMP signaling | #5278 |
| GGTACCTCTAGAAAAAGGTCATCTGATAAAAGGTCACTCGAAAAAAGGTCATCTGATAAAAGGTCACTCGAGCCTGCAGG | | |
| **NFAT**, [27] | $Ca^{2+}$-signaling | #5343 |
| GGTACCTCTAGAGGATTTTCCATCTGATGGATTTTCCACTCGAAGGATTTTCCATCTGATGGATTTTCCACTCGAGCCTGCAGG | | |
| **NFAT/AP1**, [28] | $Ca^{2+}$-signaling | #5305 |
| GGTACCTCTAGATGGAAAATTTGACTCATAGTCTGATTGGAAAATTTGACTCATAGCTCGAATGGAAAATTTGACTCATAGTCTGATTGGAAAATTTGACTCATAGCTCGAGCCTGCAGG | | |
| **NFkappaB**, [29] | Rel-domain transcription factors | #5264 |
| GGTACCTCTAGAGGGAATTCCCTCTGATGGGAATTCCCCTCGAAGGGAATTCCCTCTGATGGGAATTCCCCTCGAGCCTGCAGG | | |
| **NFY**, [3] | CCAAT transcription factors | #5292 |
| GGTACCTCTAGAAGCCAATCGGTCTGATAGCCAATCGGCTCGAAAGCCAATCGGTCTGATAGCCAATCGGCTCGAGCCTGCAGG | | |
| **NRF**, [3] | Reactive oxygen species (ROS) signaling | #5294 |
| GGTACCTCTAGACTGCGCATGCGCTCTGATCTGCGCATGCGCCTCGAACTGCGCATGCGCTCTGATCTGCGCATGCGCCTCGAGCCTGCAGG | | |
| **Pea3b**, [30] | FGF signaling | #5272 |

| | | |
|---|---|---|
| GGTACCTCTAGAGGAAATTCCTTTCCTCTGATGGAAATTCCTTTCCCTCGAAGGAAATTCCTTTCCTCTGATGGAAATTCCTTTCCCTCGAGCCTGCAGG | | |
| **RBP-J**, [31,32] | Notch signaling | #5268 |
| GGTACCTCTAGACGTGGGAATCTGATCGTGGGAACTCGAACGTGGGAATCTGATCGTGGGAACTCGAGCCTGCAGG | | |
| **Runx**, JASPAR database | Runx transcription factors | #5307 |
| GGTACCTCTAGACTGTGGTTTTCTGATCTGTGGTTTCTCGAACTGTGGTTTTCTGATCTGTGGTTTCTCGAGCCTGCAGG | | |
| **SBE**, [32,33] | TGFβ signaling | #5276 |
| GGTACCTCTAGAAGACAGACATCTGATAGCCAGACACTCGAAAGACAGACATCTGATAGCCAGACACTCGAGCCTGCAGG | | |
| **SF-1**, [24] | steroid signaling | #5279 |
| GGTACCTCTAGATCAAGGTCATCTGATTCAAGGTCACTCGAATCAAGGTCATCTGATTCAAGGTCACTCGAGCCTGCAGG | | |
| **Sim2**, [34] | Sim transcription factors | #5344 |
| GGTACCTCTAGATTGTTATGCAAATCTGATTTGTTATGCAAACTCGAATTGTTATGCAAATCTGATTTGTTATGCAAACTCGAGCCTGCAGG | | |
| **Sox/Pou**, [35] | Pou-domain transcription factors | #5299 |
| GGTACCTCTAGACATTGACATGCTAATTCTGATCATTGACATGCTAATCTCGAACATTGACATGCTAATTCTGATCATTGACATGCTAATCTCGAGCCTGCAGG | | |
| **SP1/KLF**, [3] | SP/KLF transcription factors | #5293 |
| GGTACCTCTAGAGGCCCCGCCCCCTCTGATGGCCCCGCCCCCCTCGAAGGCCCCGCCCCCTCTGATGGCCCCGCCCCCCTCGAGCCTGCAGG | | |
| **SRE**, [36] | SRF signaling | #5280 |
| GGTACCTCTAGACCATAAAAGGTCTGATCCATATAAGGCTCGAACCATAAAAGGTCTGATCCATATAAGGCTCGAGCCTGCAGG | | |
| **STAT3**, [37–39] | STAT3-signaling | #5289 |
| GGTACCTCTAGAATTTCCCGGAAATTCTGATATTTCCCGGAAATCTCGAAATTTCCCGGAAATTCTGATATTTCCCGGAAATCTCGAGCCTGCAGG | | |
| **T-box**, [40] | T-box transcription factors | #5345 |
| GGTACCTCTAGATCACACCTTCTGATTCACACCTCTCGAATCACACCTTCTGATTCACACCTCTCGAGCCTGCAGG | | |
| **TCF/LEF**, [41,42] | Wnt-signaling | #5274 |
| GGTACCTCTAGAAGATCAAAGGGTCTGATAGATCAAAGGGCTCGAAAGATCAAAGGGTCTGATAGATCAAAGGGCTCGAGCCTGCAGG | | |
| **TEAD**, [43,44] | YAP/TAZ-signaling | #5392 |
| GGTACCTCTAGAACATTCCACTCTGATACATTCCACCTCGAAACATTCCACTCTGATACATTCCACCTCGAGCCTGCAGG | | |

**Table S28:** List of Firefly luciferase constructs containing 4 transcription factor DNA binding sites. The abbreviation of the respective binding sites are marked in bold letters. References indicate publications from which DNA consensus sequences for transcription factor binding sites were retrieved. The respective sense oligonucleotides used for cloning are shown below with transcription factor binding sites marked in red and the flanking restriction enzyme sites for *Kpn*I at the 5'-end and *Sbf*I at the 3'-end marked in green. Vector numbers relate to plasmid stock numbers in the lab internal plasmid database.

**Table S29:** List of Firefly luciferase constructs containing 2-8 transcription factor DNA binding sites

| DNA-binding site (below: sense oligo used for cloning) | Cloning procedure | Construct number |
|---|---|---|
| 2x ETS bindings sites | Oligos were annealed in cloned into *Kpn*I and *Pst*I restriction sites of pBTolmin-E1b-Firefly luciferase SV40pA | #5462 |
| GGTACCTCTAGACACTTCCGGTTCTGATCACTTCCGGTCTCGAGCCTGCAGG | | |
| 6x ETS bindings sites | Oligos were annealed and cloned downstream of 4xETS-sites (vector #5301 see TableS1 above) into *Xho*I and *Pst*I restriction sites in front of the E1b basal promoter, recombination in bacteria resulted in 6x ETS sites | #5478 |
| GTCGACCACTTCCGGTTCTGATCACTTCCGGTCTCGAACACTTCCGGTTCTGATCACTTCCGGTCCTGCAGG | | |
| 8x ETS bindings sites | Oligos were annealed and cloned downstream of 4xETS-sites (vector #5301 see TableS1 above) into *Xho*I and *Pst*I restriction sites in front of the E1b basal promoter, | #5477 |
| GTCGACCACTTCCGGTTCTGATCACTTCCGGTCTCGAACACTTCCGGTTCTGATCACTTCCGGTCCTGCAGG | | |

**Table S29:** List of Firefly luciferase constructs containing 2-8 ETS transcription factor DNA binding sites. The respective sense oligonucleotides used for cloning are shown below with transcription factor binding sites marked in red and the flanking restriction enzyme sites for *Kpn*I at the 5'-end and *Sbf*I at the 3'-end marked in green. Vector numbers relate to plasmid stock numbers in the lab internal plasmid database.

## References


1. Du, X.; Wang, J.; Zhu, H.; Rinaldo, L.; Lamar, K.-M.; Palmenberg, A.C.; Hansel, C.; Gomez, C.M. Second cistron in CACNA1A gene encodes a transcription factor mediating cerebellar development and SCA6. *Cell* **2013**, *154*, 118–133.

2. Oikawa, D.; Akai, R.; Tokuda, M.; Iwawaki, T. A transgenic mouse model for monitoring oxidative stress. *Sci. Rep.* **2012**, *2*, 229.

3. Webb, A.E.; Kundaje, A.; Brunet, A. Characterization of the direct targets of FOXO transcription factors throughout evolution. *Aging Cell* **2016**, *15*, 673–685.

4. Morikawa, M.; Koinuma, D.; Tsutsumi, S.; Vasilaki, E.; Kanki, Y.; Heldin, C.-H.; Aburatani, H.; Miyazono, K. ChIP-seq reveals cell type-specific binding patterns of BMP-specific Smads and a novel binding motif. *Nucleic Acids Res.* **2011**, *39*, 8712–8727.

5. Bobinet, M.; Vignard, V.; Florenceau, L.; Lang, F.; Labarriere, N.; Moreau-Aubry, A. Overexpression of meloe gene in melanomas is controlled both by specific transcription factors and hypomethylation. *PLoS One* **2013**, *8*, e75421–e75421.

6. Moore, S.P.G.; Kruchten, J.; Toomire, K.J.; Strauss, P.R. Transcription Factors and DNA Repair Enzymes Compete for Damaged Promoter Sites. *J. Biol. Chem.* **2016**, *291*, 5452–5460.

7. Di Vona, C.; Bezdan, D.; Islam, A.B.M.M.K.; Salichs, E.; López-Bigas, N.; Ossowski, S.; de la Luna, S. Chromatin-wide Profiling of DYRK1A Reveals a Role as a Gene-Specific RNA Polymerase II CTD Kinase. *Mol. Cell* **2015**, *57*, 506–520.

8. Wolfe, M.W.; Call, G.B. Early Growth Response Protein 1 Binds to the Luteinizing Hormone-β Promoter and Mediates Gonadotropin-Releasing Hormone-Stimulated Gene Expression. *Mol. Endocrinol.* **1999**, *13*, 752–763.

9. Brown, L.A.; Yang, S.-H.; Hair, A.; Galanis, A.; Sharrocks, A.D. Molecular characterization of a zebrafish TCF ETS-domain transcription factor. *Oncogene* **1999**, *18*, 7985–7993.

10. Boros, J.; Donaldson, I.J.; O'Donnell, A.; Odrowaz, Z.A.; Zeef, L.; Lupien, M.; Meyer, C.A.; Liu, X.S.; Brown, M.; Sharrocks, A.D. Elucidation of the ELK1 target gene network reveals a role in the coordinate regulation of core components of the gene regulation machinery. *Genome Res.* **2009**, *19*, 1963–1973.

11. Odrowaz, Z.; Sharrocks, A.D. ELK1 uses different DNA binding modes to regulate functionally distinct classes of target genes. *PLoS Genet.* **2012**, *8*, e1002694–e1002694.

12. Gorelick, D.A.; Halpern, M.E. Visualization of estrogen receptor transcriptional activation in zebrafish. *Endocrinology* **2011**, *152*, 2690–2703.

13. Chen, L.; Zheng, J.; Yang, N.; Li, H.; Guo, S. Genomic selection identifies vertebrate transcription factor Fezf2 binding sites and target genes. *J. Biol. Chem.* **2011**, *286*, 18641–18649.

14. Fischer, J.A.; Giniger, E.; Maniatis, T.; Ptashne, M. GAL4 activates transcription in Drosophila. *Nature* **1988**, *332*, 853–856.

15. Köster, R.W.; Fraser, S.E. Tracing Transgene Expression in Living Zebrafish Embryos. *Dev. Biol.* **2001**, *233*, 329–346.

16. Kinzler, K.W.; Vogelstein, B. The GLI gene encodes a nuclear protein which binds specific sequences in the human genome. *Mol. Cell. Biol.* **1990**, *10*, 634–642.

17. Sasaki, H.; Hui, C.; Nakafuku, M.; Kondoh, H. A binding site for Gli proteins is essential for HNF-3beta floor plate enhancer activity in transgenics and can respond to Shh in vitro. *Development* **1997**, *124*, 1313 LP – 1322.

18. Kulkarni, R.P.; Tohari, S.; Ho, A.; Brenner, S.; Venkatesh, B. Characterization of a hypoxia-response element in the Epo locus of the pufferfish, Takifugu rubripes. *Mar. Genomics* **2010**, *3*, 63–70.

19. Greenald, D.; Jeyakani, J.; Pelster, B.; Sealy, I.; Mathavan, S.; van Eeden, F.J. Genome-wide mapping of Hif-1α binding sites in zebrafish. *BMC Genomics* **2015**, *16*, 923.

20. Bajoghli, B.; Aghaallaei, N.; Heimbucher, T.; Czerny, T. An artificial promoter construct for heat-inducible misexpression during fish embryogenesis. *Dev. Biol.* **2004**, *271*, 416–430.

21. Emelyanov, A.; Parinov, S. Mifepristone-inducible LexPR system to drive and control gene expression in transgenic zebrafish. *Dev. Biol.* **2008**, *320*, 113–121.

22. Hagey, D.W.; Zaouter, C.; Combeau, G.; Lendahl, M.A.; Andersson, O.; Huss, M.; Muhr, J. Distinct transcription factor complexes act on a permissive chromatin landscape to establish regionalized gene expression in CNS stem cells. *Genome Res.* **2016**, *26*, 908–917.

23. Hagey, D.W.; Klum, S.; Kurtsdotter, I.; Zaouter, C.; Topcic, D.; Andersson, O.; Bergsland, M.; Muhr, J. SOX2 regulates common and specific stem cell features in the CNS and endoderm derived organs. *PLoS Genet.* **2018**, *14*, e1007224–e1007224.

24. Wilson, T.E.; Fahrner, T.J.; Milbrandt, J. The orphan receptors NGFI-B and steroidogenic factor 1 establish monomer binding as a third paradigm of nuclear receptor-DNA interaction. *Mol. Cell. Biol.* **1993**, *13*, 5794–5804.

25. Callard, G. V; Tchoudakova, A. V; Kishida, M.; Wood, E. Differential tissue distribution, developmental programming, estrogen regulation and promoter characteristics of cyp19 genes in teleost fish. *J. Steroid Biochem. Mol. Biol.* **2001**, *79*, 305–314.

26. Zhou, F.; Drabsch, Y.; Dekker, T.J.A.; de Vinuesa, A.G.; Li, Y.; Hawinkels, L.J.A.C.; Sheppard, K.-A.; Goumans, M.-J.; Luwor, R.B.; de Vries, C.J.; et al. Nuclear receptor NR4A1 promotes breast cancer invasion and metastasis by activating TGF-β signalling. *Nat. Commun.* **2014**, *5*, 3388.

27. Zhang, W.; Takahara, T.; Achiha, T.; Shibata, H.; Maki, M. Nanoluciferase Reporter Gene System Directed by Tandemly Repeated Pseudo-Palindromic NFAT-Response Elements Facilitates Analysis of Biological Endpoint Effects of Cellular Ca(2+) Mobilization. *Int. J. Mol. Sci.* **2018**, *19*, 605.

28. Chen, L.; Glover, J.N.M.; Hogan, P.G.; Rao, A.; Harrison, S.C. Structure of the DNA-binding domains from NFAT, Fos and Jun bound specifically to DNA. *Nature* **1998**, *392*, 42–48.

29. Kuri, P.; Ellwanger, K.; Kufer, T.A.; Leptin, M.; Bajoghli, B. A high-sensitivity bi-directional reporter to monitor NF-κB activity in cell culture and zebrafish in real time. *J. Cell Sci.* **2017**, *130*, 648 LP – 657.

30. Znosko, W.A.; Yu, S.; Thomas, K.; Molina, G.A.; Li, C.; Tsang, W.; Dawid, I.B.; Moon, A.M.; Tsang, M. Overlapping functions of Pea3 ETS transcription factors in FGF signaling during zebrafish development. *Dev. Biol.* **2010**, *342*, 11–25.

31. Parsons, M.J.; Pisharath, H.; Yusuff, S.; Moore, J.C.; Siekmann, A.F.; Lawson, N.; Leach, S.D. Notch-responsive cells initiate the secondary transition in larval zebrafish pancreas. *Mech. Dev.* **2009**, *126*, 898–912.

32. Schiavone, M.; Rampazzo, E.; Casari, A.; Battilana, G.; Persano, L.; Moro, E.; Liu, S.; Leach, S.D.; Tiso, N.; Argenton, F. Zebrafish reporter lines reveal in vivo signaling pathway activities involved in pancreatic cancer. *Dis. Model. Mech.* **2014**, *7*, 883–894.

33. Dennler, S.; Itoh, S.; Vivien, D.; ten Dijke, P.; Huet, S.; Gauthier, J.M. Direct binding of Smad3 and Smad4 to critical TGF beta-inducible elements in the promoter of human plasminogen activator inhibitor-type 1 gene. *EMBO J.* **1998**, *17*, 3091–3100.

34. Letourneau, A.; Cobellis, G.; Fort, A.; Santoni, F.; Garieri, M.; Falconnet, E.; Ribaux, P.; Vannier, A.; Guipponi, M.; Carninci, P.; et al. HSA21 Single-Minded 2 (Sim2) Binding Sites Co-Localize with Super-Enhancers and Pioneer Transcription Factors in Pluripotent Mouse ES Cells. *PLoS One* **2015**, *10*, e0126475–e0126475.

35. Onichtchouk, D.; Geier, F.; Polok, B.; Messerschmidt, D.M.; Mössner, R.; Wendik, B.; Song, S.; Taylor, V.; Timmer, J.; Driever, W. Zebrafish Pou5f1-dependent transcriptional networks in temporal control of early development. *Mol. Syst. Biol.* **2010**, *6*, 354.

36. Davis, J.; Long, X.; Georger, M.; Scott, I.; Rich, A.; Miano, J. Expression and comparative genomics of two serum response factor genes in zebrafish. *Int. J. Dev. Biol.* **2008**, *52*, 389–396.

37. Langlais, D.; Couture, C.; Balsalobre, A.; Drouin, J. Regulatory network analyses reveal genome-wide potentiation of LIF signaling by glucocorticoids and define an innate cell defense response. *PLoS Genet.* **2008**, *4*, e1000224–e1000224.

38. Vallania, F.; Schiavone, D.; Dewilde, S.; Pupo, E.; Garbay, S.; Calogero, R.; Pontoglio, M.; Provero, P.; Poli, V. Genome-wide discovery of functional transcription factor binding sites by comparative genomics: the case of Stat3. *Proc. Natl. Acad. Sci. U. S. A.* **2009**, *106*, 5117–5122.

39. Salmon-Divon, M.; Dvinge, H.; Tammoja, K.; Bertone, P. PeakAnalyzer: genome-wide annotation of chromatin binding and modification loci. *BMC Bioinformatics* **2010**, *11*, 415.

40. Morley, R.H.; Lachani, K.; Keefe, D.; Gilchrist, M.J.; Flicek, P.; Smith, J.C.; Wardle, F.C. A gene regulatory network directed by zebrafish No tail accounts for its roles in mesoderm formation. *Proc. Natl. Acad. Sci. U. S. A.* **2009**, *106*, 3829–3834.

41. Shimizu, N.; Kawakami, K.; Ishitani, T. Visualization and exploration of Tcf/Lef function using a highly responsive Wnt/β-catenin signaling-reporter transgenic zebrafish. *Dev. Biol.* **2012**, *370*, 71–85.

42. Moro, E.; Ozhan-Kizil, G.; Mongera, A.; Beis, D.; Wierzbicki, C.; Young, R.M.; Bournele, D.; Domenichini, A.; Valdivia, L.E.; Lum, L.; et al. In vivo Wnt signaling tracing through a transgenic biosensor fish reveals novel activity domains. *Dev. Biol.* **2012**, *366*, 327–340.

43. Davidson, I.; Xiao, J.H.; Rosales, R.; Staub, A.; Chambon, P. The HeLa cell protein TEF-1 binds specifically and cooperatively to two SV40 enhancer motifs of unrelated sequence. *Cell* **1988**, *54*, 931–942.

44. Miesfeld, J.B.; Link, B.A. Establishment of transgenic lines to monitor and manipulate Yap/Taz-Tead activity in zebrafish reveals both evolutionarily conserved and divergent functions of the Hippo pathway. *Mech. Dev.* **2014**, *133*, 177–188.
